# Analytic integrability of generalized 3-dimensional chaotic systems

**DOI:** 10.1371/journal.pone.0302062

**Published:** 2024-04-25

**Authors:** Ahmad Muhamad Husien, Azad Ibrahim Amen

**Affiliations:** 1 Department of Mathematics, College of Science, University of Duhok, Duhok, Kurdistan Region, Iraq; 2 Department of Mathematics, College of Basic Education, Salahaddin University-Erbil, Erbil, Kurdistan Region, Iraq; 3 Department of Mathematics, Faculty of Science, Soran University, Soran, Kurdistan Region, Iraq; 4 Department of Mathematics, Basic Education College, Raparin University, Ranya, Kurdistan Region, Iraq; Lanzhou University of Technology, CHINA

## Abstract

Numerous recently introduced chaotic systems exhibit straightforward algebraic representations. In this study, we explore the potential for identifying a global analytic first integral in a generalized 3-dimensional chaotic system (2). Our work involves detailing the model of a new 3-D chaotic system characterized by three Lyapunov exponents—positive, zero, and negative. We depict the phase trajectories, illustrate bifurcation patterns, and visualize Lyapunov exponent graphs. The investigation encompasses both local and global analytic first integrals for the system, providing results on the existence and non-existence of these integrals for different parameter values. Our findings reveal that the system lacks a global first integral, and the presence or absence of analytic first integrals is contingent upon specific parameter values. Additionally, we present a formal series for the system, demonstrating 3D and 2D projections of the system (2) for a given set of initial conditions achieved by selecting alternative values for parameters *a*, *b*, *c*, *d*, *r* and *l*.

## 1 Introduction

The literature has shown significant interest in chaotic circuits due to their applications in various fields such as secure communications, robotics, image processing, and random bit generation. The electrical engineer’s foundational understanding revolves around linear circuit theory, serving as a benchmark in their circuit-related considerations. When confronted with nonlinear circuits, they perceive their behavior as an altered rendition of linear circuit behavior. In this context, signal distortion, harmonic generation, and similar effects stem evidently from the nonlinear attributes of circuit elements. Utilizing series expansions to analyze deviations from linear behavior becomes an intuitive and customary approach for investigating these phenomena [1]. Adaptive oscillators can learn and encode information in dynamic, plastic states. In [2] the pendulum has recently been proposed as the base oscillator of an adaptive system. In a mechanical setup, the horizontally forced pendulum adaptive frequency oscillator seeks a resonance condition by modifying the length of the pendulum’s rod. In this paper [3], focuses on the nonlinear generalized Calogero–Bogoyavlenskii–Schiff equation to explain the wave profiles in soliton theory. The improved and efficient technique is applied to derive soliton solutions that are dependent, significant, and more broadly applicable for this equation, surpassing the intricacy of prior complex travel equations. The objective of this research [4] is to investigate the nonlinear Landau–Ginzburg–Higgs equation, which characterizes nonlinear solitary waves exhibiting distant and feeble scattering interactions among tropical tropospheres and mid-latitudes. While Table I in [5] encompasses conservative systems, the main focus lies on dissipative systems due to their tendency to produce more resilient electrical circuits. In the realm of electrical circuits, evading dissipation parallels the construction of a mechanical system devoid of friction. An issue arises from these circuits having a region beyond which their dynamics become unbounded, leading to op amps reaching saturation. To address op amp saturation, circuit restarts or discharge of charge from capacitors become necessary. Furthermore, ensuring op amps possess a relatively high slew rate is crucial. Apart from these concerns, no obstacles were encountered during the construction of any circuits. Notably, at audio frequencies, concerns such as stray capacitance and inductance posed no problems, and there were no instances of parasitic oscillations. The initial chaotic circuit was developed by Chua [6] and has found use in chaos-based generators and other applications. Subsequently, additional diverse chaotic electronic circuits, including simple RLC and RC circuits [7–9], oscillators [10, 11], and capacitor circuits, were introduced. In [12], then electronic circuit design of the new chaotic system was implemented considering practical applications. An autonomous system with a dimension of three or higher, contingent on the parameters, can demonstrate chaotic behavior under certain parameter configurations while possessing first integrals under different parameter settings. This phenomenon is notably exemplified by the Lorenz system and Rössler systems [13–16]. In general, establishing whether a specific system is chaotic or possesses first integrals can be challenging.

The main contributions of this paper to system (2) can be listed as follows:

1. We have described the mathematical model of a new 3-D general autonomous chaotic system (2) from the system (1) and compared its Lyapunov exponents and Kaplan-Yorke dimension with the recent system (2).
2. We have presented a detailed bifurcation analysis of the proposed system (2) using bifurcation diagrams and Lyapunov exponents (LEs) and observed nonlinear phenomena like a self-excited, a hidden attractor, and chaotic behavior.
3. We have carried out simulations of the proposed system (2) using an electronic circuit designed via MultiSim.
4. We have implemented the proposed system (2) in FPGA and showed experimental attractors observed in an oscilloscope to verify their chaotic behavior.
5. The final contribution of this work is the finding of non-existence types of first integrals for the system (2).

## 2 Modelling of chaotic system

In [17], announces a novel three-dimensional chaotic system with line equilibrium and discusses its dynamic properties such as Lyapunov exponents, phase portraits, equilibrium points, bifurcation diagram, multistability and coexisting attractors. We also display the implementation of the Field-Programmable Gate Array (FPGA) based Pseudo-Random Number Generator (PRNG) by using the new chaotic system. In [18], a hyperjerk system pertains to a dynamical system regulated by an ordinary differential equation of nth order, where *n* ≥ 4. Also, in [19], we describe the model of a new 5-D hyperchaotic system with three positive Lyapunov exponents. Since the maximum positive Lyapunov exponent of the proposed hyperchaotic system is larger than twelve, the new hyperchaotic system is highly hyperchaotic. We also show that the new 5-D hyperchaotic system exhibits multistability with coexisting attractors.

In their work [20], the authors introduced a quadratic chaotic system with self-excited and hidden attractors, described by the following equations, dependent on the real parameters *a*, *b*, and *c*:

$$\begin{array}{c} \dot{x}=y+b,\;\;\;\dot{y}=-7x+4yz,\;\;\;\dot{z}=a-x^{2}-y^{2}+cxz. \end{array}$$ (1)

The dynamic properties of system (1) were extensively investigated through numerical simulations in [20]. Additionally, the authors implemented the system as an electronic circuit to demonstrate real-time engineering applications.

In this study, we present a generalized version of system (1), denoted as (2), with the following equations:

$$\begin{array}{c} \dot{x}=y+b,\;\;\;\dot{y}=dx+ryz,\;\;\;\dot{z}=a+lx^{2}+ly^{2}+cxz. \end{array}$$ (2)

Here, *a*, *b*, *c* are real parameters, and it is required that *drl* ≠ 0. Interestingly, a similar system was considered in [21], given by:

$$\begin{array}{c} \begin{array}{c} \dot{x}=y,\;\;\;\;\dot{y}=-x+yz,\;\;\;\dot{z}=-x-axy-bxz. \end{array} \end{array}$$ (3)

The aforementioned study [21] focused on the dynamical analysis of system (3) at infinity and limit cycles. Our analysis encompasses invariant algebraic surfaces, exponential factors, and investigates the integrability and non-integrability of system (3).

## 3 Preliminary results

In this section, you will find condensed summaries of the integrability problem, analytic first integrals, and supplementary outcomes. Furthermore, fundamental definitions and theorems are provided to substantiate the primary findings of the study.

**Definition 1**. [22–24] *An attractor is classified as **self-excited** if its basin of attraction intersects with any open neighborhood of a stationary state, recognized as an equilibrium. Conversely, if such an intersection does not occur, the attractor is termed **a hidden** attractor*.

**Definition 2**. [25] *Suppose U is an open subset of* $R^{3}$. *A non-constant function* F:U→R *is considered a first integral of the polynomial system* (2) *on U if it remains constant along the orbits* (*x*(*t*), *y*(*t*), *z*(*t*)) *of* (2) *with in U*. *In other words, F*(*x*(*t*), *y*(*t*), *z*(*t*)) *is constant for all values of t*. *The function F is classified as a first integral of* (2) *on U if and only if the following equation is satisfied*:

$$\begin{array}{c} \left(y+b\right)\frac{\partial F}{\partial x}+\left(dx+ryz\right)\frac{\partial F}{\partial y}+\left(a+lx^{2}+ly^{2}+cxz\right)\frac{\partial F}{\partial z}=0, \end{array}$$ (4)

*on U*.

*Consequently, F remains invariant along every trajectory curve, and if F is an analytic function, it is regarded as an analytic first integral*.

**Definition 3**. [13] *The total energy F is deemed a formal first integral if it can be expressed as a formal series expansion in the vicinity of the singular point* (*x*_0_, *y*_0_, *z*_0_).

**Definition 4**. [26] *A global first integral for the system* (2) *refers to a first integral that is applicable across the entire domain* $R^{3}$.

**Definition 5**. [26] *A local first integral for the system* (2) *is a first integral defined with in a neighborhood of an equilibrium point of the system* (2).

**Theorem 1**. [27] *If there are no polynomial first integrals for the linear part of the system* (2) *in the vicinity of the equilibrium point* (*x*_0_, *y*_0_, *z*_0_), *it implies that there are no analytic first integrals for the entire system in a neighborhood of* (*x*_0_, *y*_0_, *z*_0_).

**Theorem 2**. [28, 29] *If the system* (2) *possesses an isolated singular point* (*x*_0_, *y*_0_, *z*_0_) *that acts as either an attractor or a repellor, then there are no C*^1^-*first integrals defined in the vicinity of* (*x*_0_, *y*_0_, *z*_0_).

**Theorem 3**. [30, 31] *The system of three-dimensional linear equations, represented as*

$$\left(\begin{array}{c} \dot{x}\\ \dot{y}\\ \dot{z} \end{array}\right)=P\left(\begin{array}{c} x\\ y\\ z \end{array}\right),$$

*where P is a matrix, possesses two distinct first integrals, denoted as F*_1_ *and F*_2_. *The expressions for these first integrals are provided in the following cases*:

**Case 1**. $F_{1}=\frac{x^{2}}{2\;xz-y^{2}}$ *and* $F_{2}=xe^{-\frac{ly}{x}}$ *if* $P=(\begin{array}{ccc} l & 0 & 0\\ 1 & l & 0\\ 0 & 1 & l \end{array})$, *with* l∈R\{0}.
**Case 2**. $F_{1}=\frac{{\left(x^{2}+y^{2}\right)}^{l}}{z^{2\;\alpha }}$ *and* $F_{2}={\left(x^{2}+y^{2}\right)}^{\beta }e^{-2\;\alpha \;\text{arctan}(\frac{y}{x})}$ *if* $P=(\begin{array}{ccc} \alpha & -\beta & 0\\ \beta & \alpha & 0\\ 0 & 0 & l \end{array})$, *with* l,α,β∈R\{0}.
**Case 3**. *F*_1_ = *x*^2^ + *y*^2^ *and* $F_{2}=\;z^{\beta }e^{-\;l\;\text{arctan}(\frac{y}{x})}$ *if* $P=(\begin{array}{ccc} 0 & -\beta & 0\\ \beta & 0 & 0\\ 0 & 0 & l \end{array})$, *with* l,β∈R\{0}.

**Theorem 4 (Routh-Hurwitz criterion)**. [32] *The negativity of the real parts of all the roots of the characteristic polynomial’s zero, expressed as L*(λ) = λ^3^ + *a*_1_λ^2^ + *a*_2_λ + *a*_3_ = 0, *is a necessary and sufficient condition. This condition is met when the coefficients satisfy a*_1_ > 0, *a*_2_ > 0, *a*_3_ > 0 *and a*_1_*a*_2_ − *a*_3_ > 0.

**Theorem 5**. [33] *Consider an analytic differential system* (2) *defined in a neighborhood of the origin in* $R^{3}$, *where the origin serves as a singularity. Let* λ_1_, λ_2_, λ_3_ *represent the eigenvalues of the linear part of the system at the origin. We define the set S as follows*:

$$\begin{array}{c} S=\{\;\left(k_{1},k_{3},k_{3}\right)\in Z_{+}^{3}:\sum_{i=1}^{3}k_{i}l_{i}=0,\;\sum_{i=1}^{3}k_{i}>0\;\}. \end{array}$$

*Suppose that the differential system* (2) *possesses r* < 3 *functionally independent analytic first integrals F*_1_, …, *F_r_ in a neighborhood of the origin. If the* R-linear *space spanned by S has a dimension of r*, *then any non-trivial analytic first integral of the system in the neighborhood of the origin can be expressed as an analytic function of F*_1_, …, *F_r_*.

**Theorem 6**. [34, 35] *Let’s assume that the eigenvalues* λ_1_, λ_2_, λ_3_ *of the Jacobian matrix satisfy* λ_1_ = 0 *and k*_2_λ_2_ + *k*_3_λ_3_ ≠ 0 *for any* $k_{2},k_{3}\in Z^{+}⋃\left\{0\right\}$ *with* *k*_2_ + *k*_3_ ≥ 1. *In such a scenario, the system* (2), *possesses a formal series first integral in a neighborhood of* (0, 0, 0) *if and only if the singular point* (0, 0, 0) *is not isolated. However, if the singular point* (0, 0, 0) *is indeed isolated, the system* (2) *lacks an analytic first integral in a neighborhood of* (0, 0, 0).

**Theorem 7**. [36] *Consider the polynomial differential system* (2). *Let’s assume that* λ_1_ = 0, λ_2_ *and* λ_3_ *are eigenvalues of Jacobian matrix at origin. In this case, the system* (2) *possesses an analytic first integral in a neighborhood of* (0, 0, 0) *if and only if the singular point* (0, 0, 0) *is not isolated*.

## 4 Results and their proofs

### 4.1 Dynamic properties of the new system

**Fig 1:**
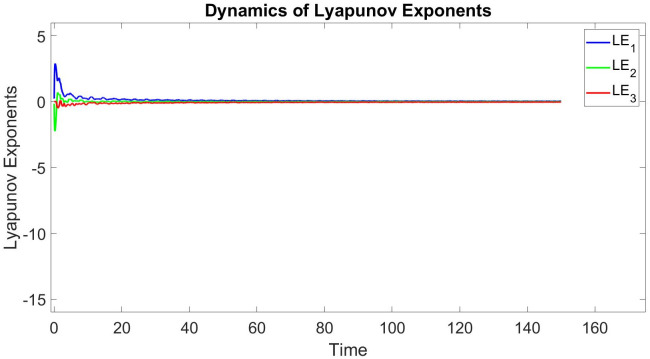
Plot of Lyapunov exponents for system (2) for initial conditions [0.1, 0.01, 0.01], when *a* = 0.8, *b* = 0, *c* = 0.01, *d* = −7.4, *r* = 6 and *l* = −2.

**Fig 2:**
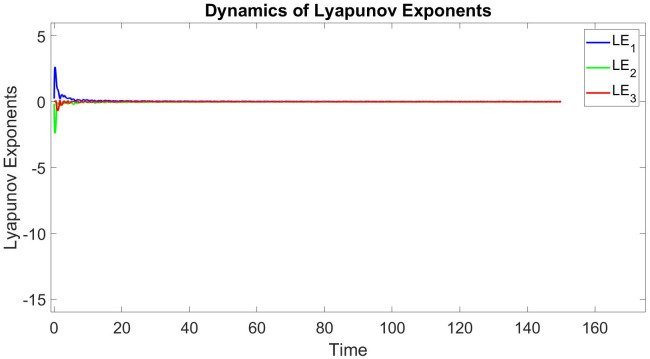
Plot of Lyapunov exponents for system (2) for initial conditions [0.1, 0.01, 0.01], when *a* = 0.3, *b* = 0.2, *c* = 0.3, *d* = −7.4, *r* = 6 and *l* = −2.

**Fig 3:**
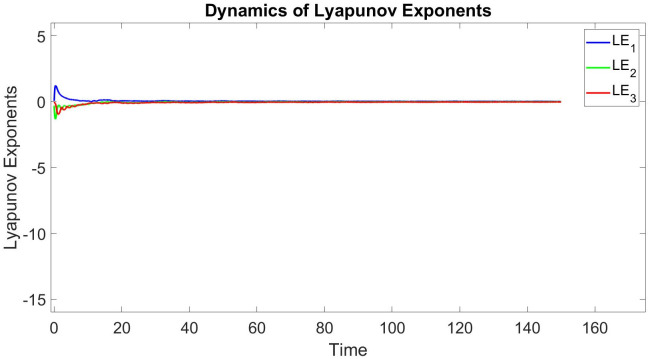
Plot of Lyapunov exponents for system (2) for initial conditions [0.1, −0.03, −0.06], when *a* = 0.05, *b* = 0.04, *c* = −0.03, *d* = −4, *r* = 4 and *l* = −5.

**Fig 4:**
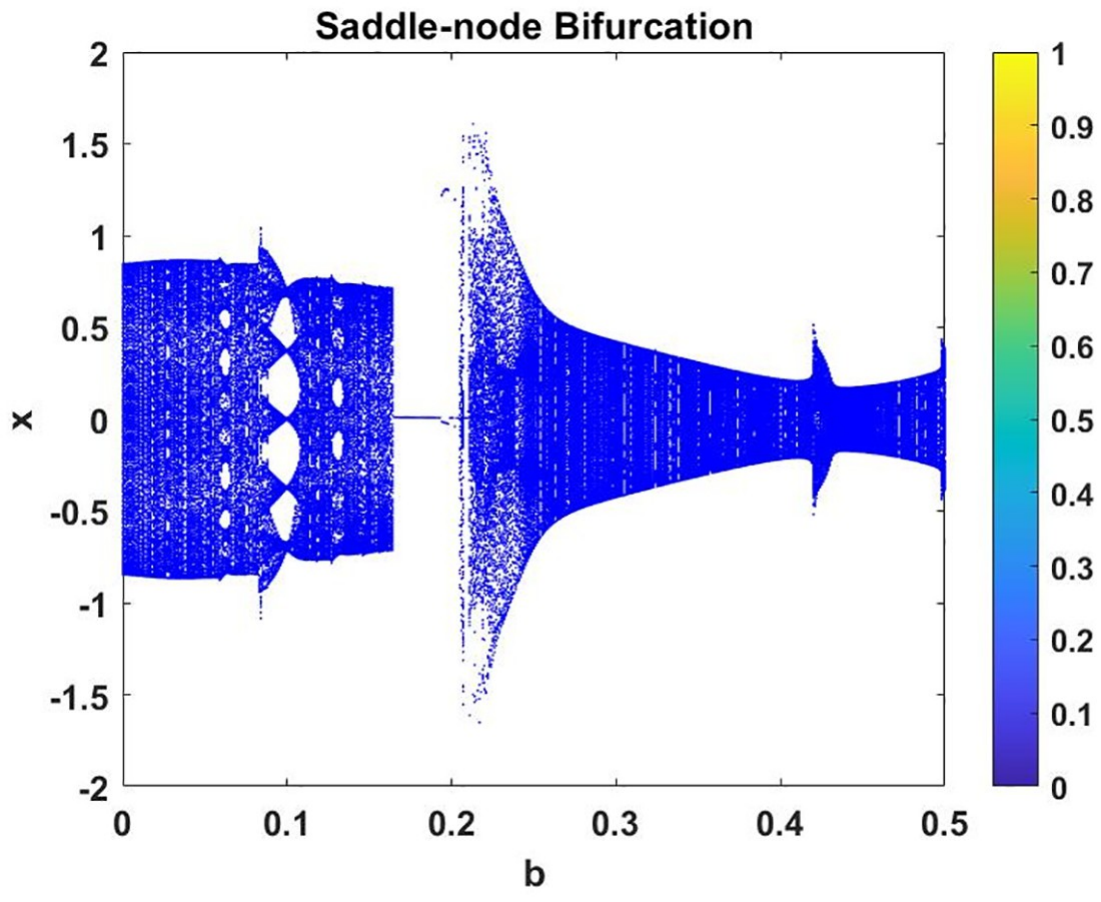
Plot of Bifurcation diagram for system (2) for initial conditions [0.1, 0.01, 0.01], when *b* ∈ [0, 0.5] *a* = 0.8, *c* = 0.01, *d* = −7.4, *r* = 6 and *l* = −2.

**Fig 5:**
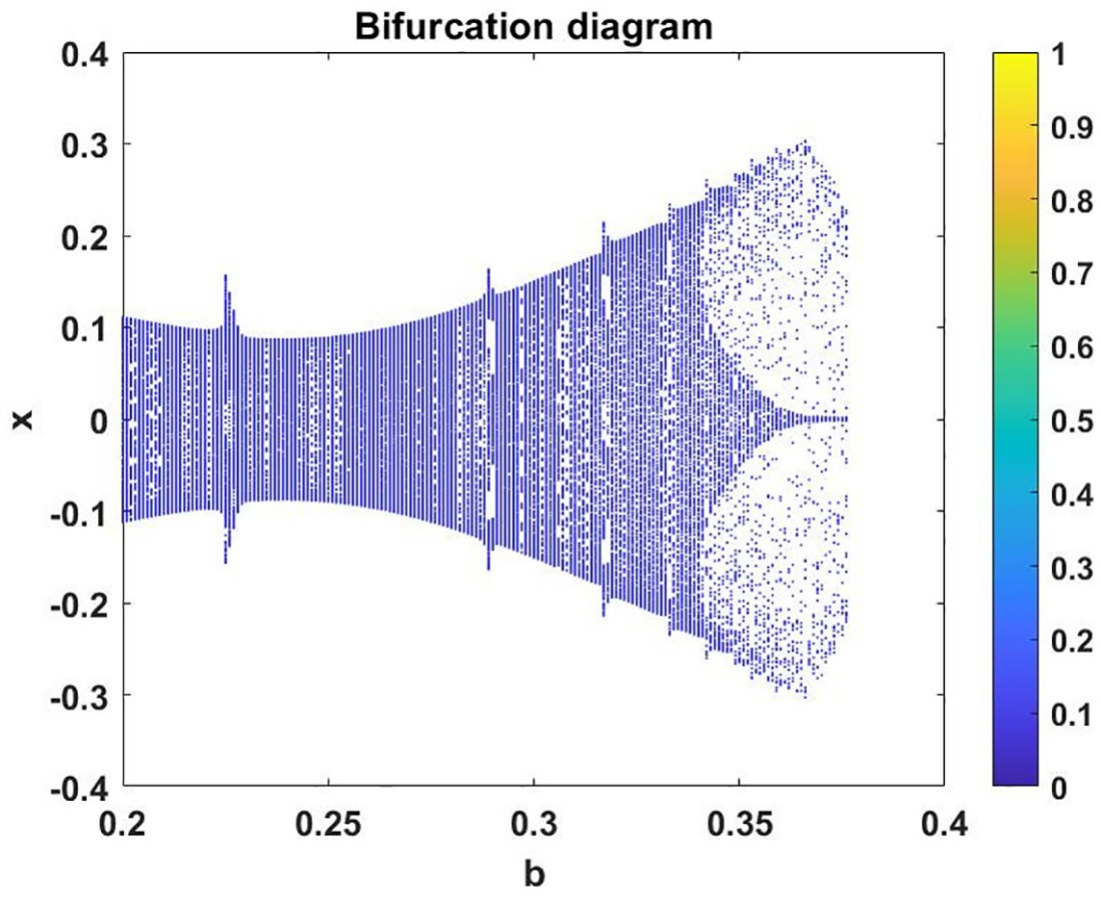
Plot of Bifurcation diagram for system (2) for initial conditions [0.1, 0.01, 0.01], when *b* ∈ [0.2, 0.3760] *a* = 0.3, *c* = 0.3, *d* = −7.4, *r* = 6 and *l* = −2.

**Fig 6:**
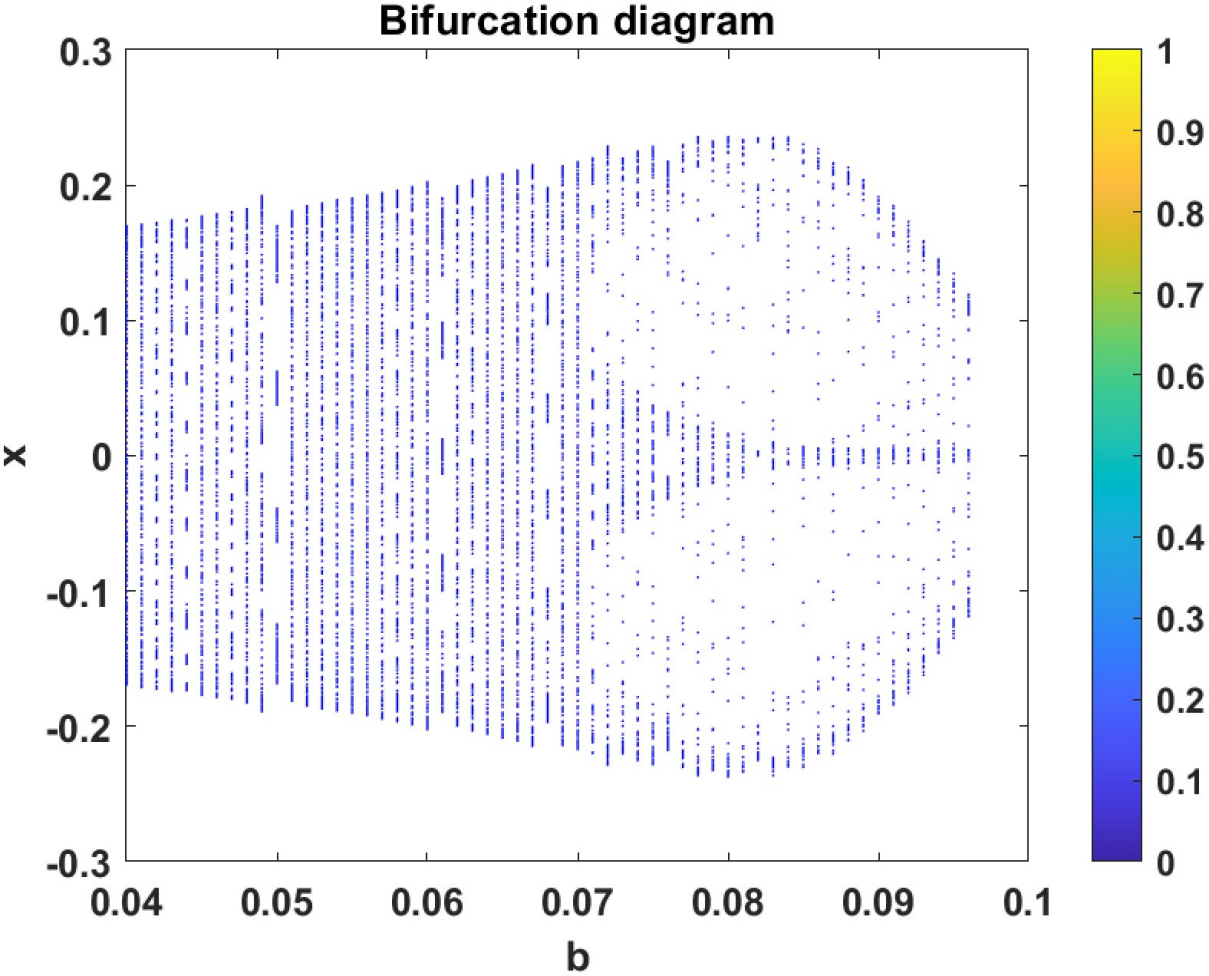
Plot of Bifurcation diagram for system (2) for initial conditions [0.1, −0.03, −0.06], when *b* ∈ [0.04, 0.3560] *a* = 0.05, *c* = −0.03, *d* = −4, *r* = 4 and *l* = −5.

In this subsection, using Matlab, we calculated the Lyapunov exponents and Bifurcation diagram of the 3-D system (2) for the initial conditions state [0.1,0.01,0.01] and [0.1,-0.03,-0.06], respectively. By choosing a different value for each of the parameters *a*, *c*, *d*, *r* and *l*. (see figures, Figs 1–6).

Where as the Lyapunov exponent measures the average predictability of a dynamical system, the dimension of its attractor measures its complexity. A fractional dimension can be defined as in [17–19, 37, 38].

$$\begin{array}{c} D_{\text{KY}}=D+\frac{\sum_{j=1}^{D}{\text{LE}}_{j}}{|{\text{LE}}_{D+1}|}. \end{array}$$ (5)

Lyapunov exponents and the Kaplan-Yorke dimension of the 3-D system (2) is calculated as follows Table 1.

**Table 1:** A comparison of Lyapunov exponents and Kaplan-Yorke dimension of three recently reported 3-D chaotic systems (2).

*a*	*b*	*c*	*d*	*r*	*l*	*LE*_1_	*LE*_2_	*LE*_3_	*T*	*DK_Y_*
0.8	0	0.01	−7.4	6	−2	0.040689	0	−0.026507	0.014182	3.535028483
0.3	0.2	0.3	−7.4	6	−2	0.015918	0	−0.0070495	0.0088685	4.258032485
0.05	0.04	−0.03	−4	4	−5	0.015214	0	−0.017977	0.014182	2.846303610

**Fig 7:**
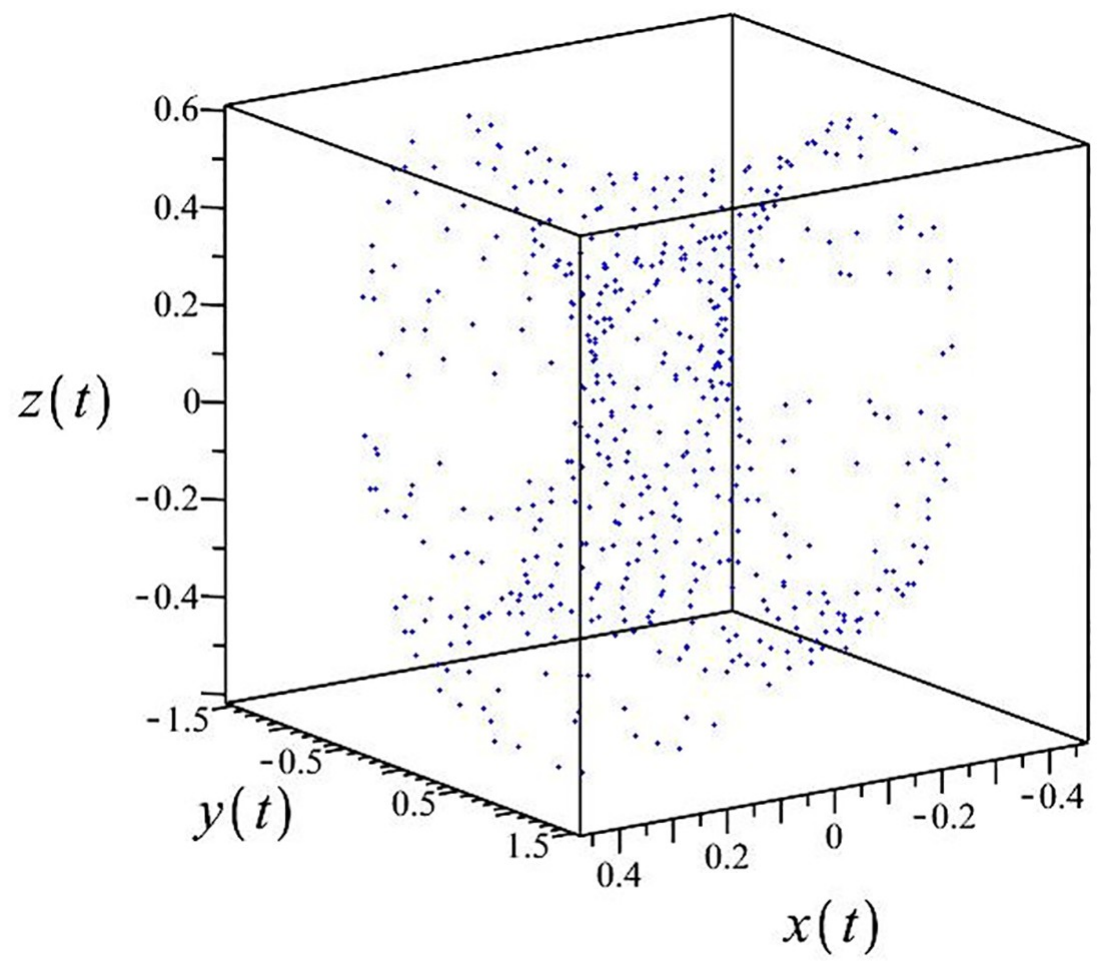
Poincaré section.

**Fig 8:**
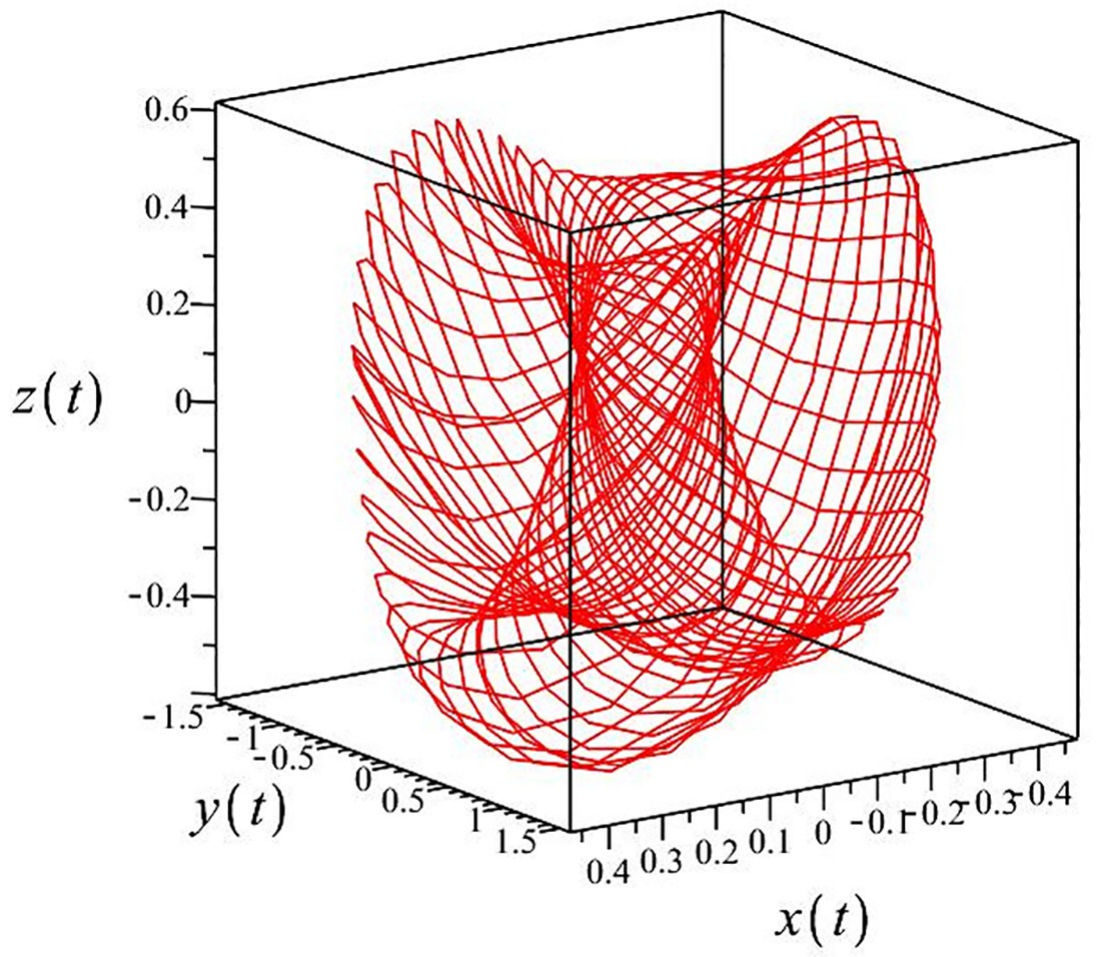
Local phase portraits of system (2) is a hidden attractor for initial conditions [0.1, 0.01, 0.01], when *a* = 0.8, *b* = 0, *c* = 0.01, *d* = −7.4, *r* = 6 and *l* = −2: On the *x*, *y*, and *z* planes, there is a 3D projection.

**Fig 9:**
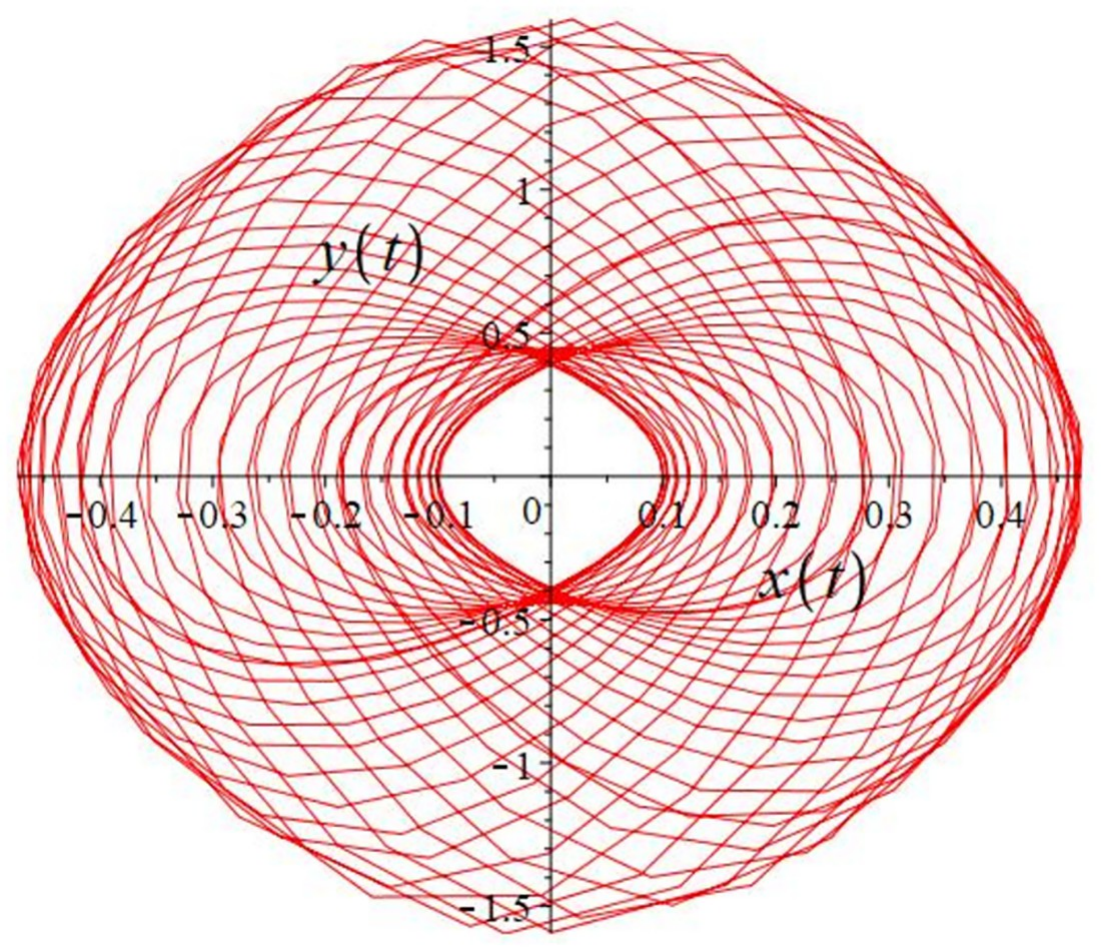
Local phase portraits of system (2) is a hidden attractor for initial conditions [0.1, 0.01, 0.01], when *a* = 0.8, *b* = 0, *c* = 0.01, *d* = −7.4, *r* = 6 and *l* = −2: On the *x*, and *y* planes, there is a 2D projection.

**Fig 10:**
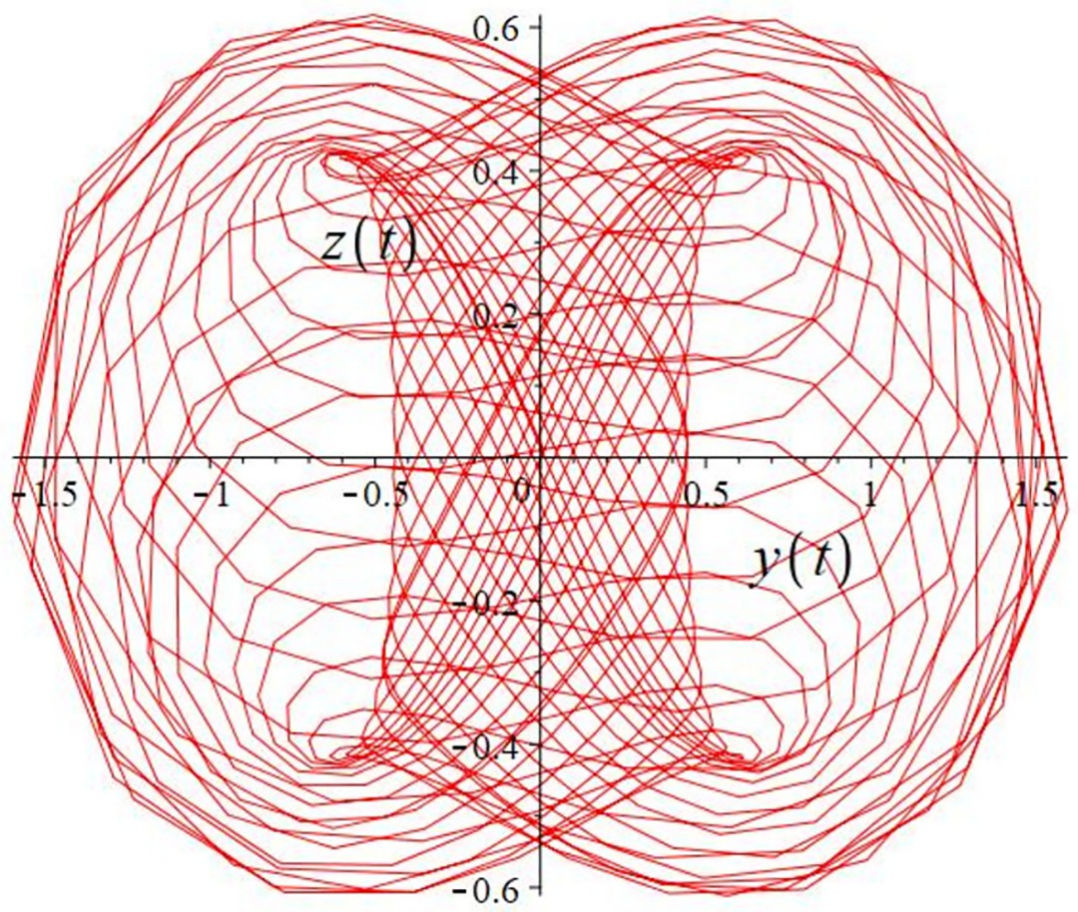
Local phase portraits of system (2) is a hidden attractor for initial conditions [0.1, 0.01, 0.01], when *a* = 0.8, *b* = 0, *c* = 0.01, *d* = −7.4, *r* = 6 and *l* = −2: On the *y*, and *z* planes, there is a 2D projection.

**Fig 11:**
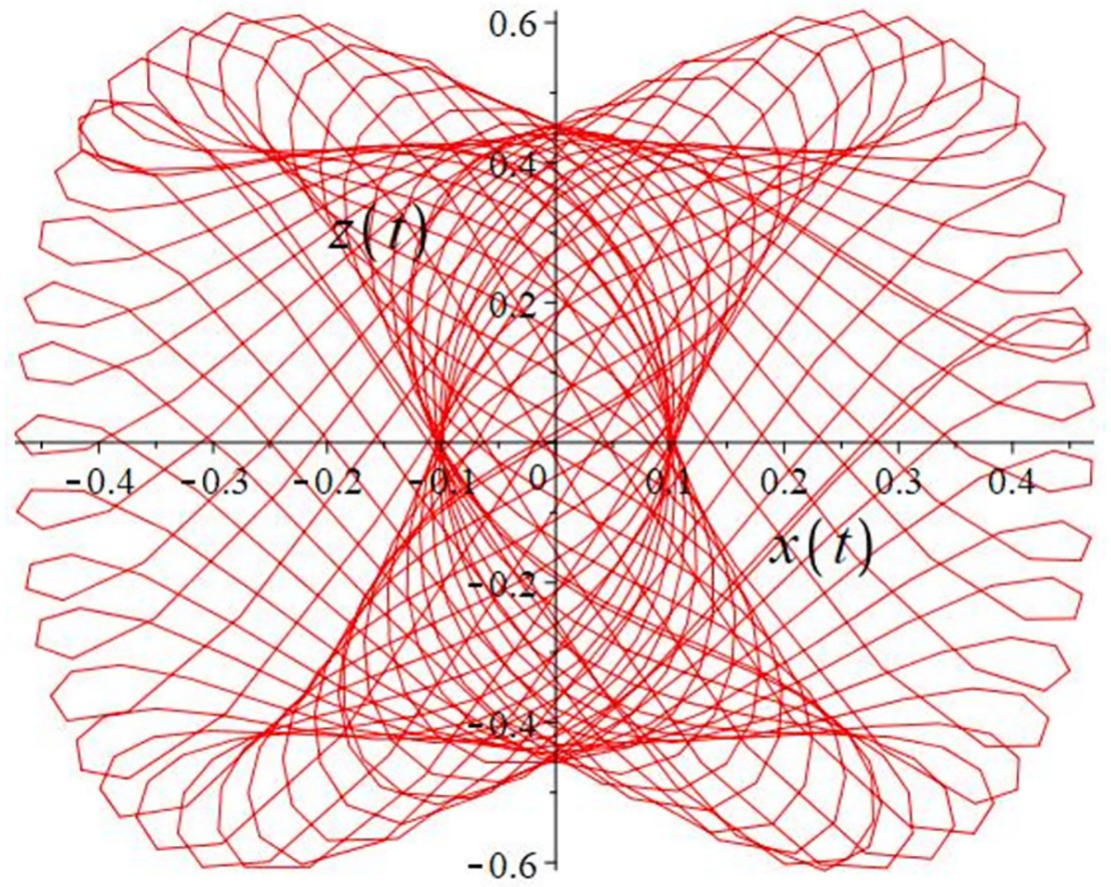
Local phase portraits of system (2) is a hidden attractor for initial conditions [0.1, 0.01, 0.01], when *a* = 0.8, *b* = 0, *c* = 0.01, *d* = −7.4, *r* = 6 and *l* = −2: On the *x*, and *z* planes, there is a 2D projection.

**Fig 12:**
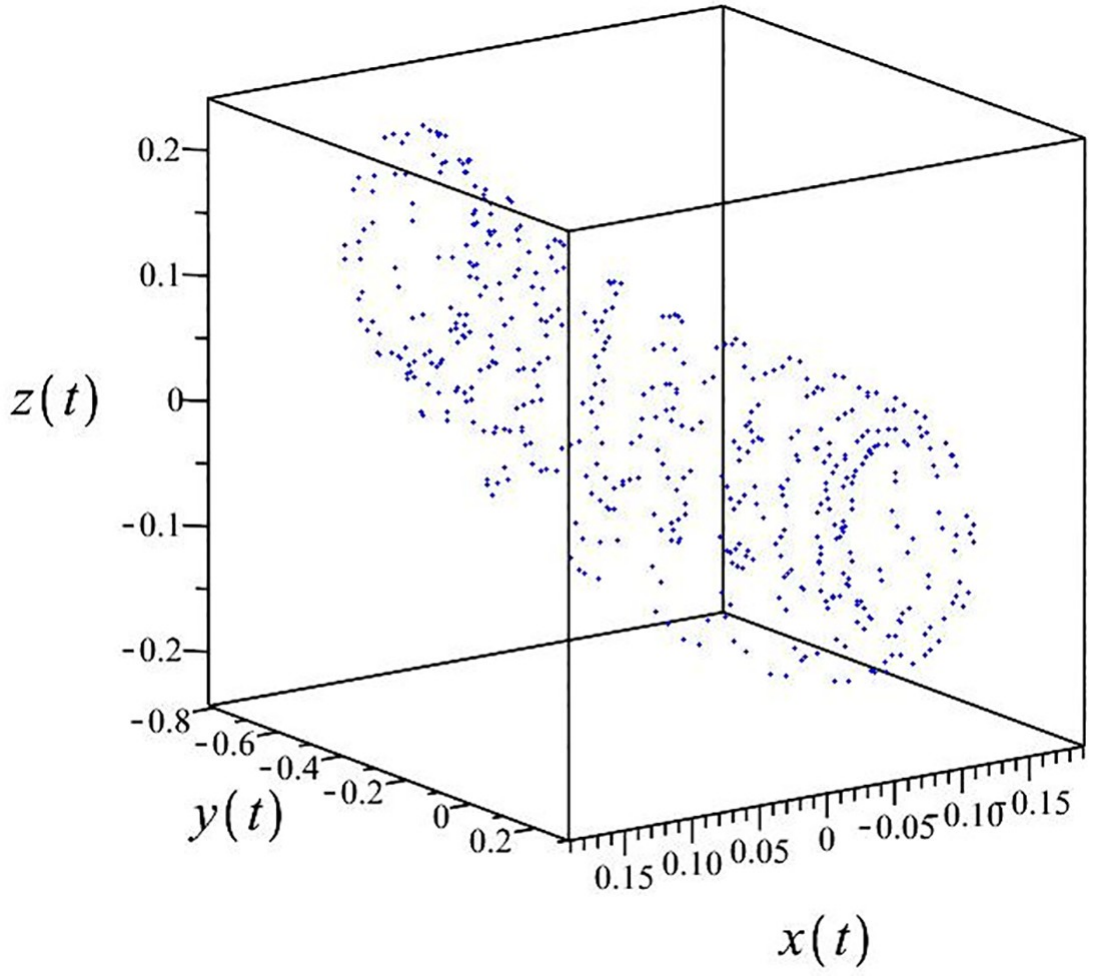
Poincaré section.

**Fig 13:**
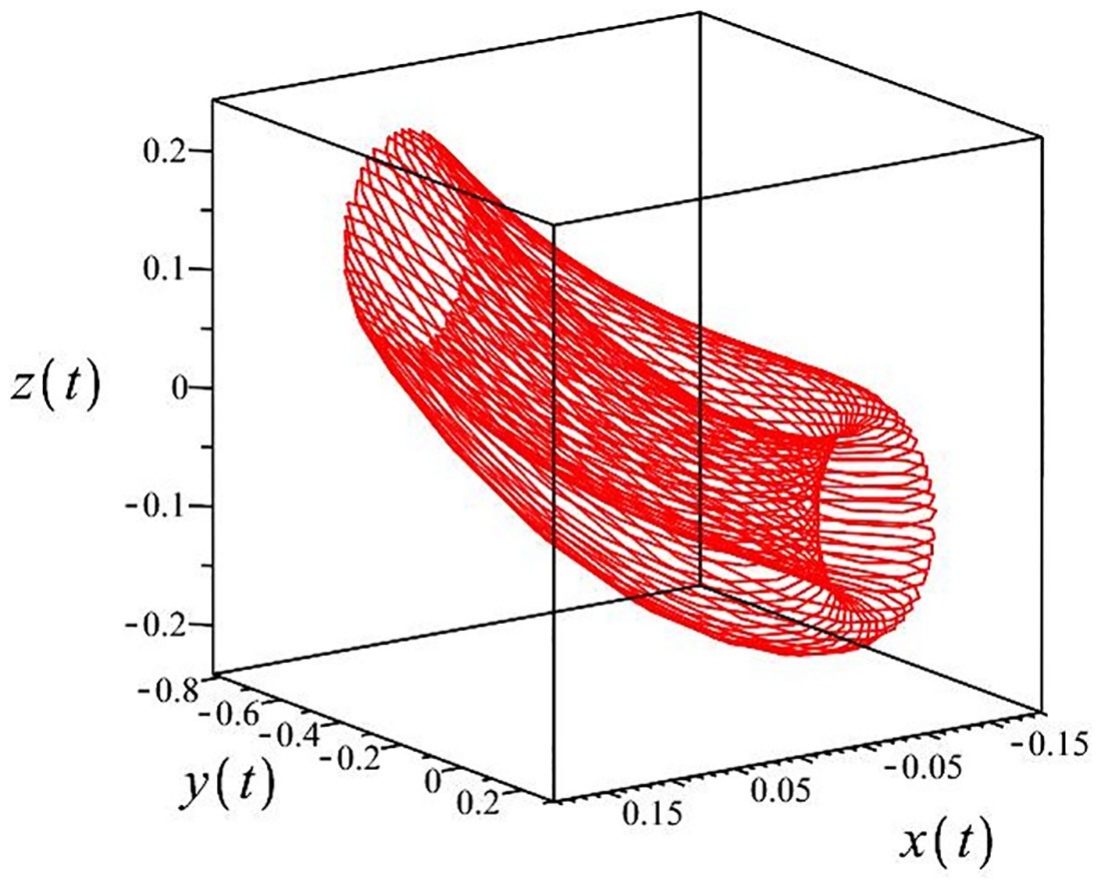
Local phase portraits of system (2) is a self-excited attractor for initial conditions [0.1, 0.01, 0.01], when *a* = 0.3, *b* = 0.2, *c* = 0.3, *d* = −7.4, *r* = 6 and *l* = −2: On the *x*, *y*, and *z* planes, there is a 3D projection.

**Fig 14:**
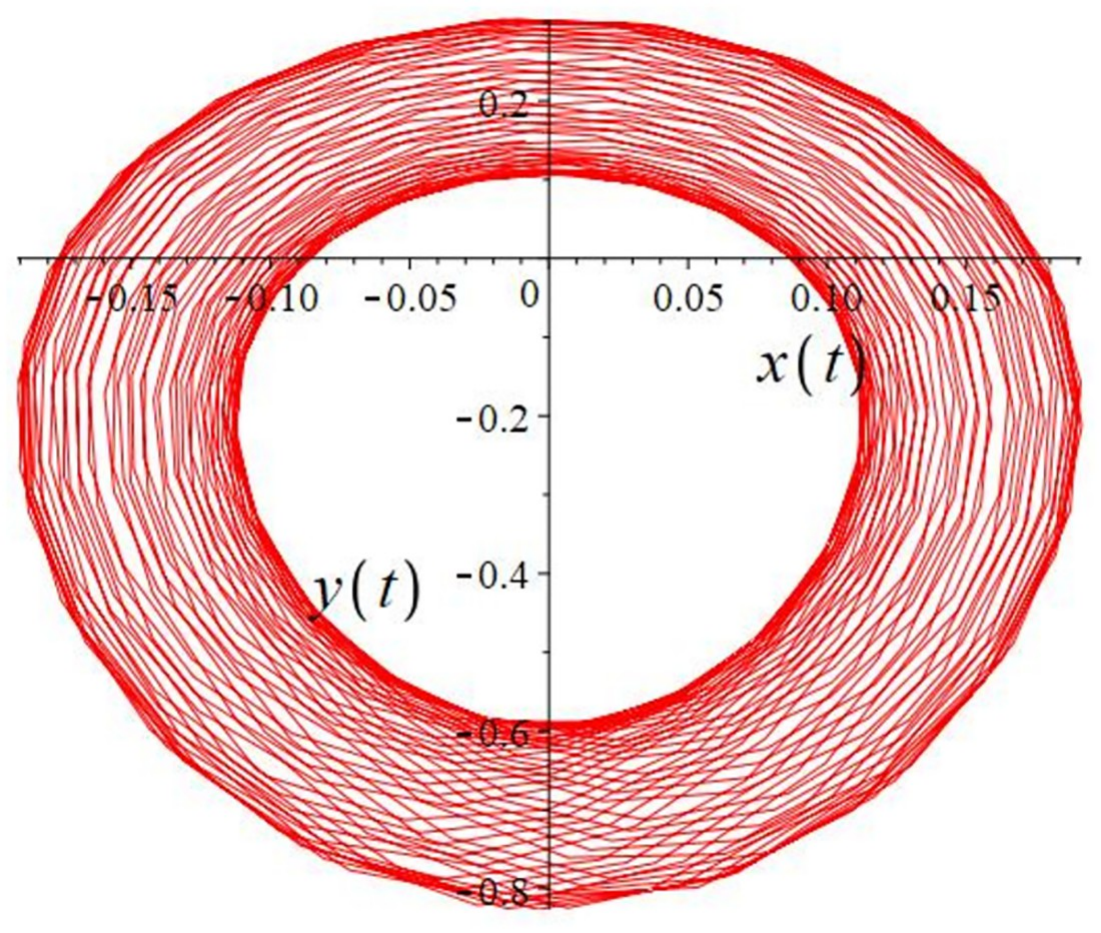
Local phase portraits of system (2) is a self-excited attractor for initial conditions [0.1, 0.01, 0.01], when *a* = 0.3, *b* = 0.2, *c* = 0.3, *d* = −7.4, *r* = 6 and *l* = −2: On the *x*, and *y* planes, there is a 2D projection.

**Fig 15:**
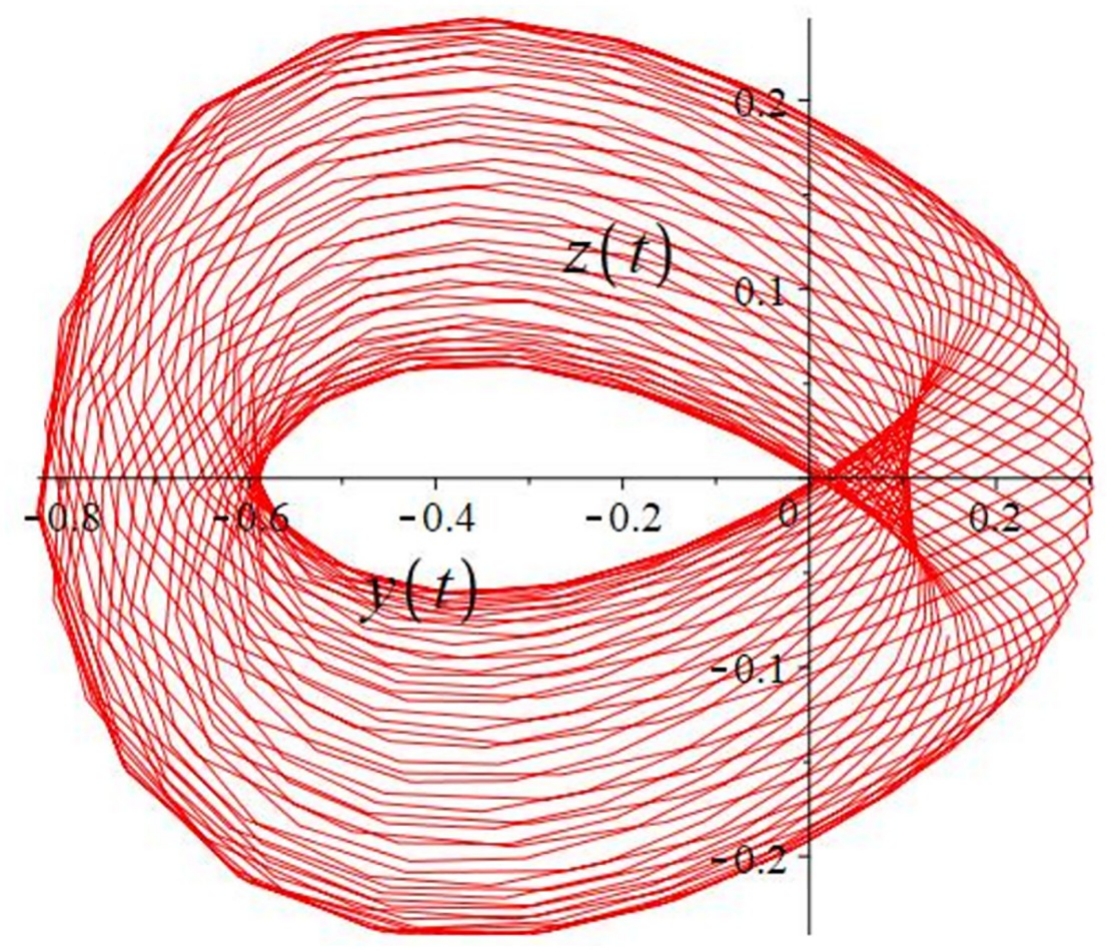
Local phase portraits of system (2) is a self-excited attractor for initial conditions [0.1, 0.01, 0.01], when *a* = 0.3, *b* = 0.2, *c* = 0.3, *d* = −7.4, *r* = 6 and *l* = −2: On the *y*, and *z* planes, there is a 2D projection.

**Fig 16:**
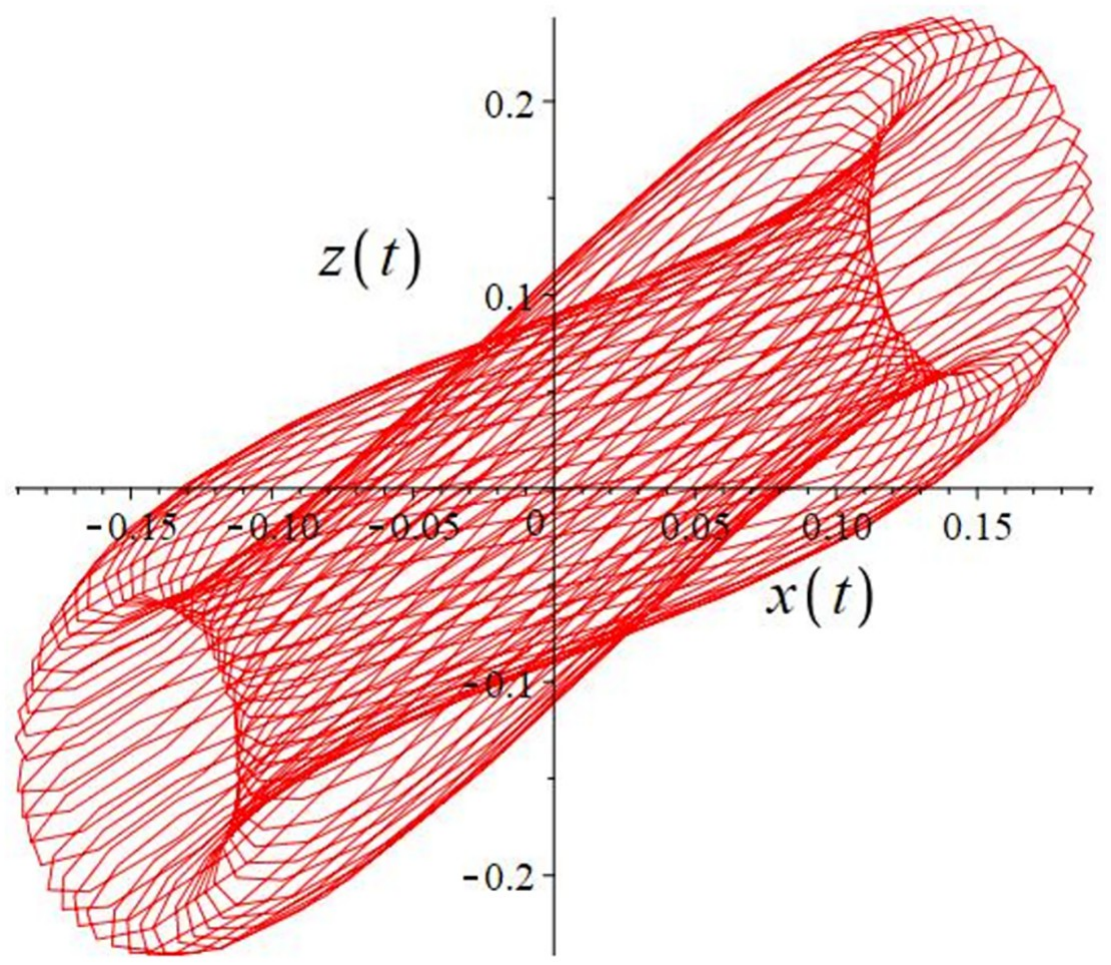
Local phase portraits of system (2) is a self-excited attractor for initial conditions [0.1, 0.01, 0.01], when *a* = 0.3, *b* = 0.2, *c* = 0.3, *d* = −7.4, *r* = 6 and *l* = −2: On the *x*, and *z* planes, there is a 2D projection.

**Fig 17:**
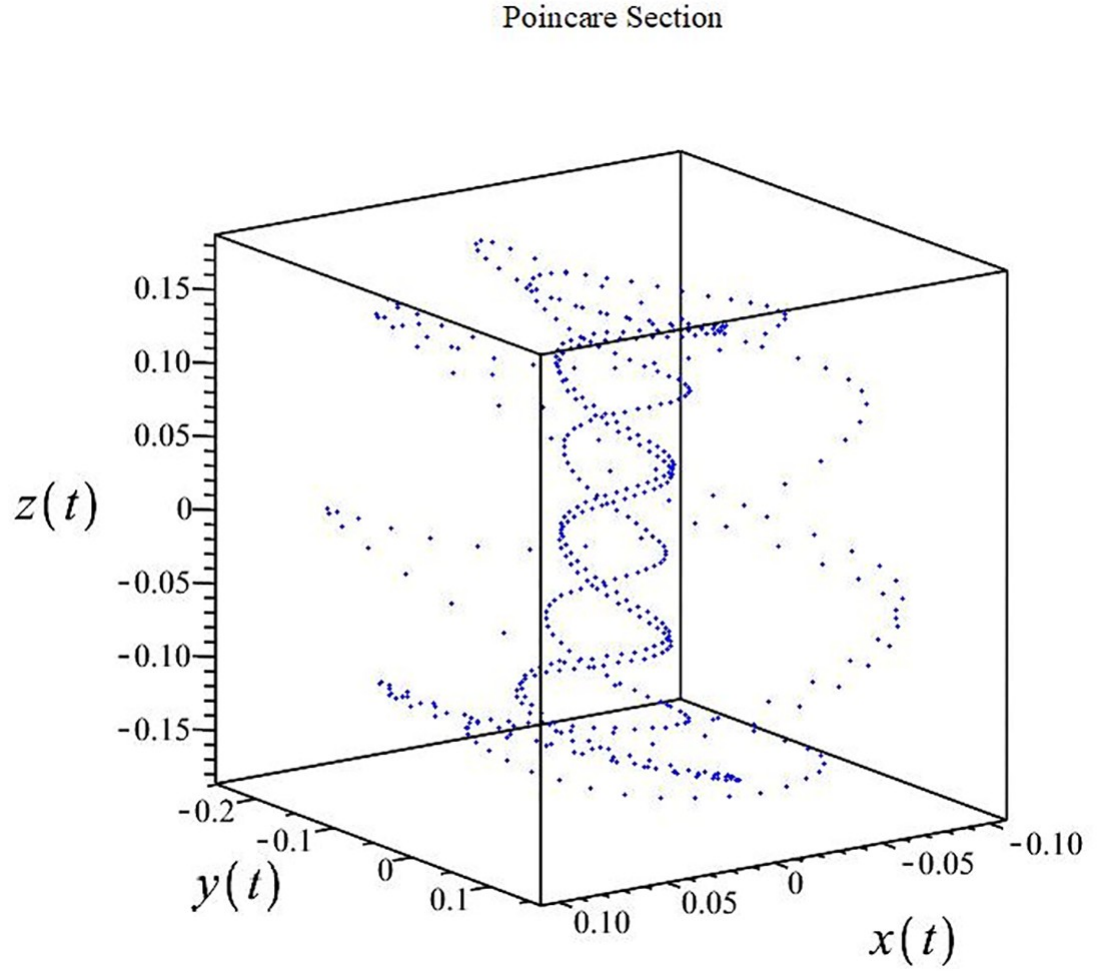
Poincaré section.

**Fig 18:**
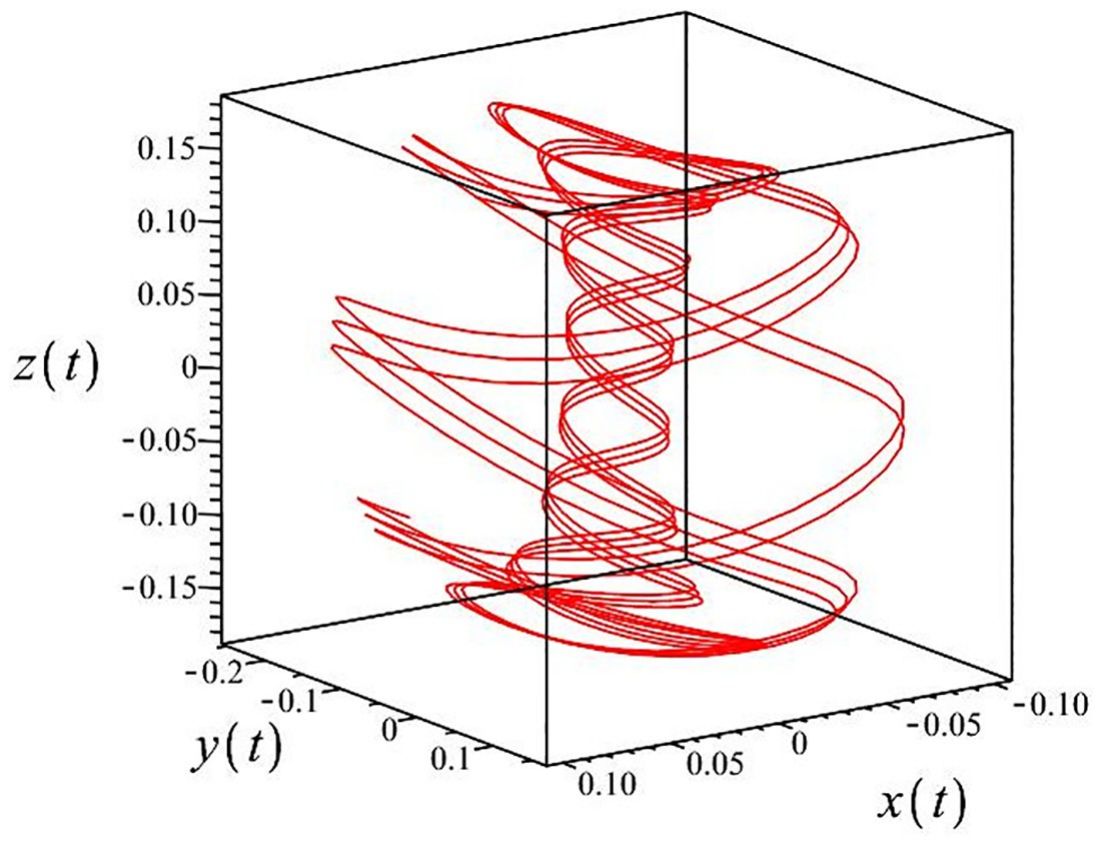
Local phase portraits of system (2) is a self-excited attractor for initial conditions [0.1, −0.03, −0.06], when *a* = 0.05, *b* = 0.04, *c* = −0.03, *d* = −4, *r* = 4 and *l* = −5: On the *x*, *y*, and *z* planes, there is a 3D projection.

**Fig 19:**
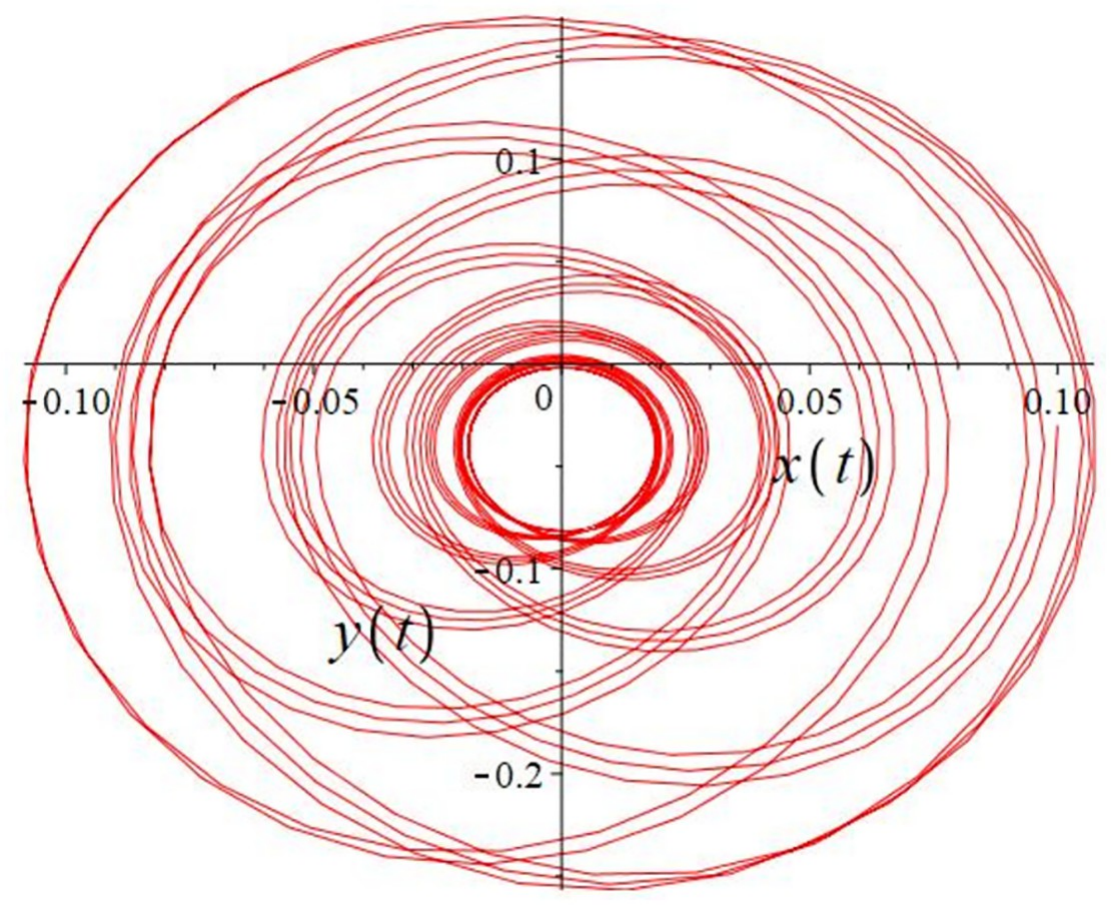
Local phase portraits of system (2) is a self-excited attractor for initial conditions [0.1, −0.03, −0.06], when *a* = 0.05, *b* = 0.04, *c* = −0.03, *d* = −4, *r* = 4 and *l* = −5: On the *x*, and *y* planes, there is a 2D projection.

**Fig 20:**
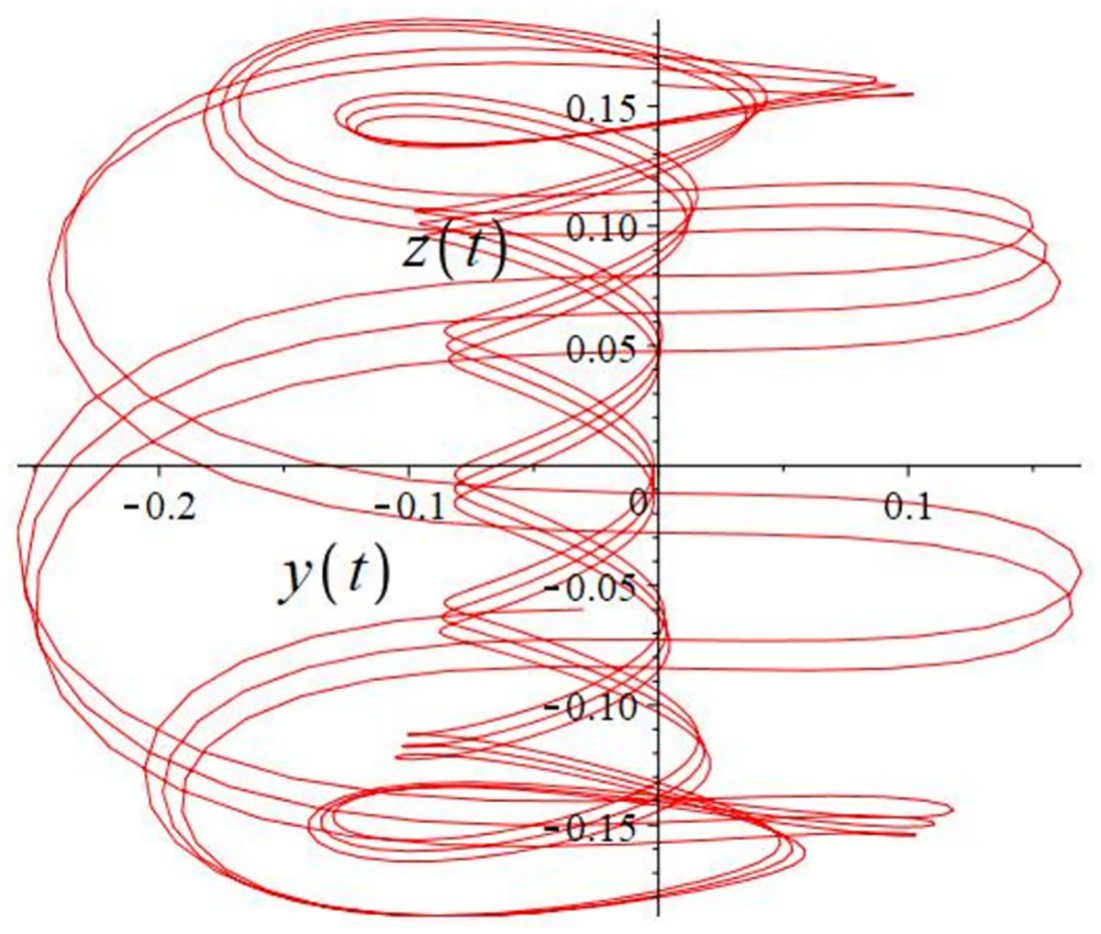
Local phase portraits of system (2) is a self-excited attractor for initial conditions [0.1, −0.03, −0.06], when *a* = 0.05, *b* = 0.04, *c* = −0.03, *d* = −4, *r* = 4 and *l* = −5: On the *y*, and *z* planes, there is a 2D projection.

**Fig 21:**
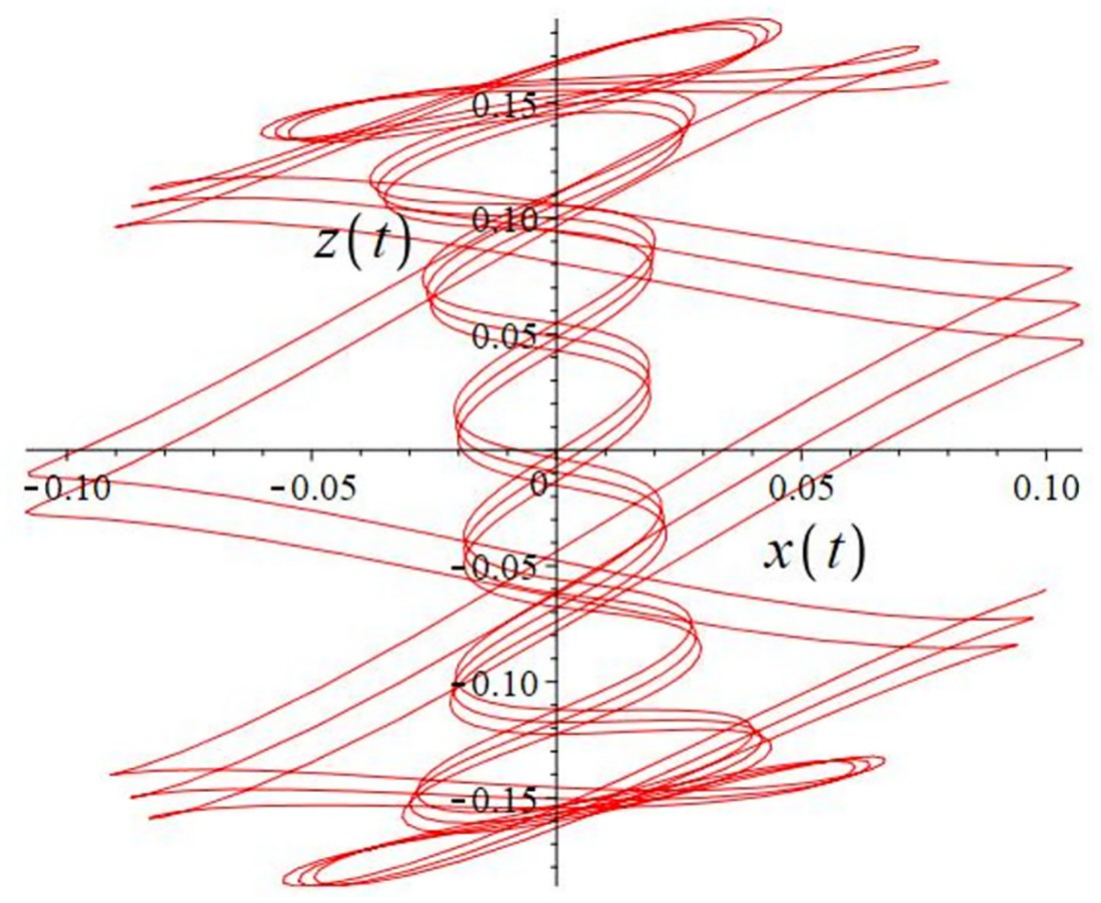
Local phase portraits of system (2) is a self-excited attractor for initial conditions [0.1, −0.03, −0.06], when *a* = 0.05, *b* = 0.04, *c* = −0.03, *d* = −4, *r* = 4 and *l* = −5: On the *x*, and *z* planes, there is a 2D projection.

**Fig 22:**
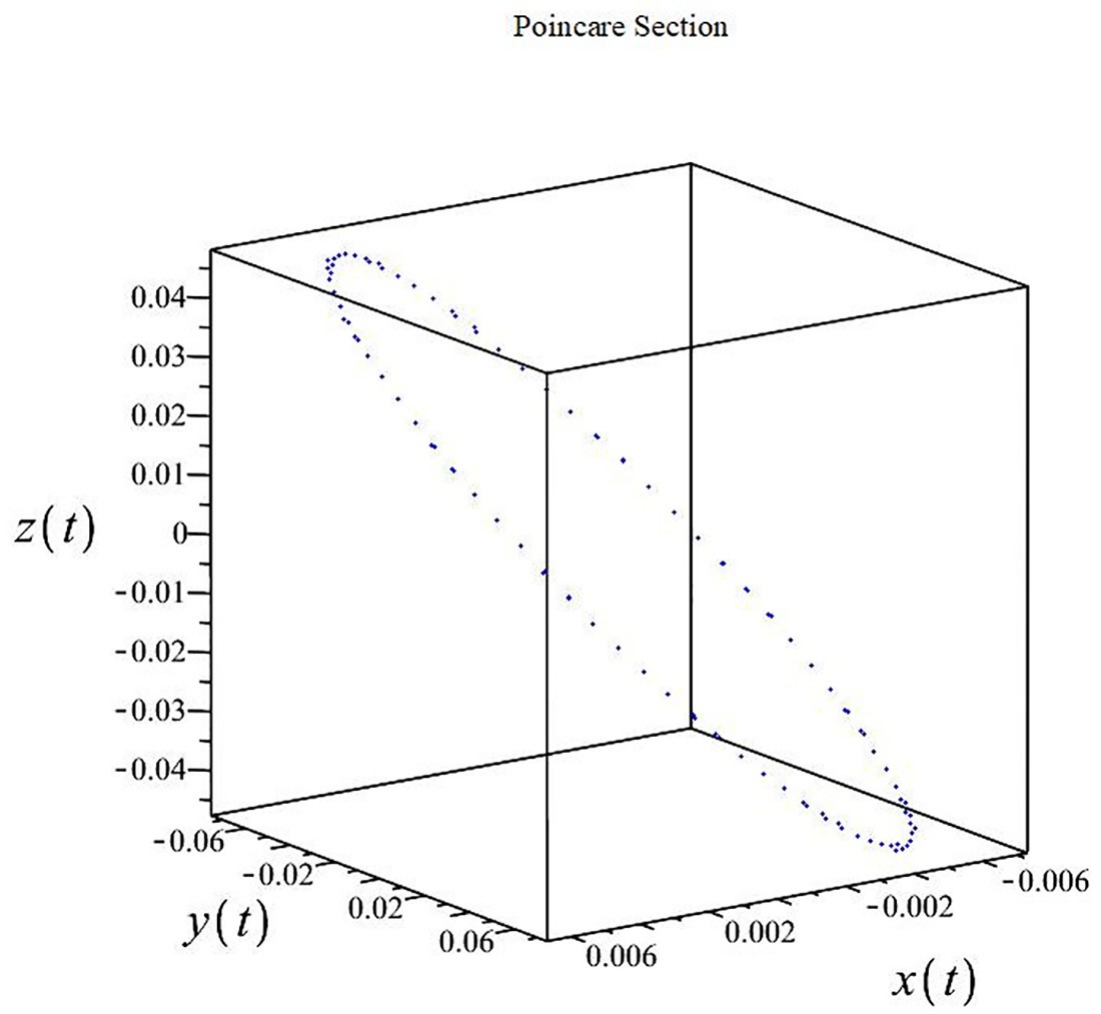
Poincaré section.

**Fig 23:**
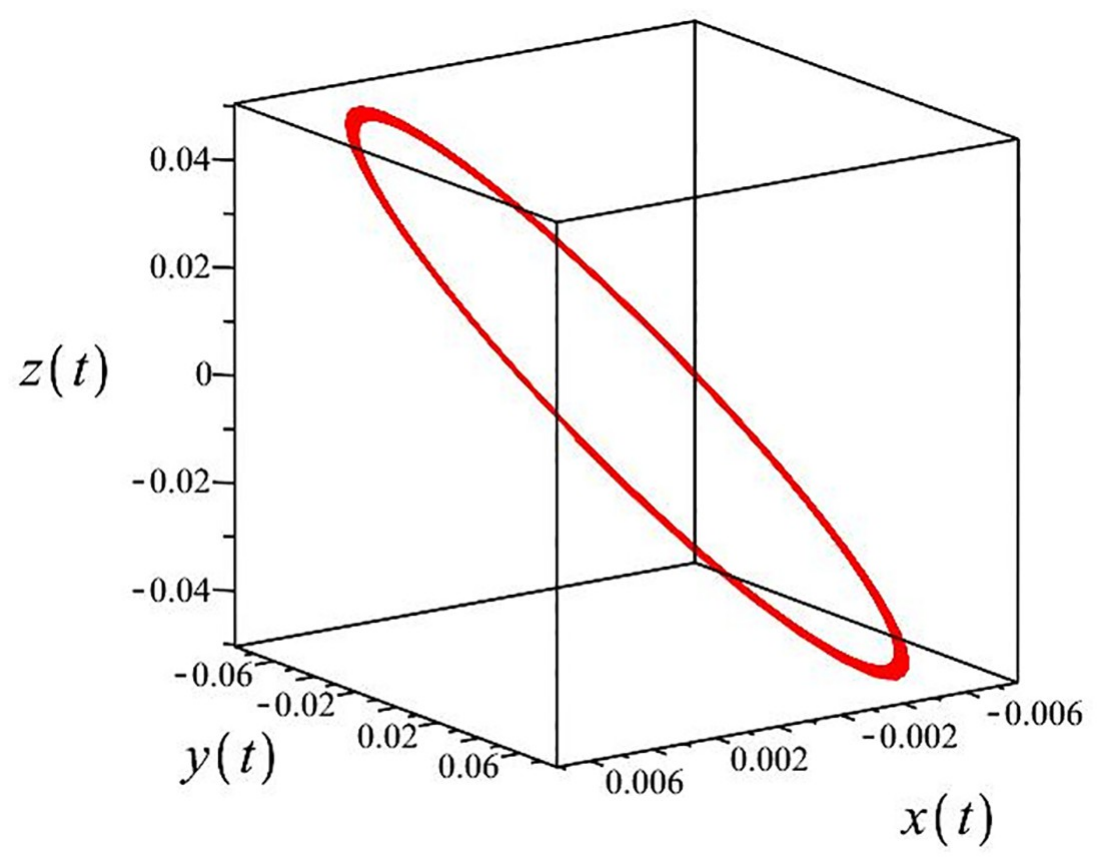
Local phase portraits of system (2) is periodic for initial conditions [0.005, 0.055, 0.005], when *a* = 1.7088, *b* = 5, *c* = 2, *d* = −7, *r* = 4 and *l* = −1: On the *x*, *y*, and *z* planes, there is a 3D projection.

The authors [20] proved that system (1) is a self-excited attractor for *a* = 0.8, *b* = 0.8, *c* = 0.01 and it is a hidden attractor for *a* = 0.8, *b* = 0, *c* = 0.01. The system (2) exhibits chaotic attractors with 3D and 2D projections. By choosing a different value for each of the parameters *a*, *c*, *d*, *r* and *l*, for a particular set of beginning conditions, 3D and 2D projections of the system (2) were plotted. The proposed system, system (2), is a self-excited attractor as parameter *b* = 0.04 and *b* = 0.2 and a hidden attractor as parameter *b* = 0. The two electronic circuit applications are implemented for different chaotic behaviours, with initial conditions [0.1,0.01,0.01] and [0.1,-0.03,-0.06], respectively. These forecasts underwent careful numerical and theoretical investigation (see figures, Figs 7–21), according to the points on the Poincaré section figures, the system is chaotic for some values of the parameters. Also, by the Poincaré section, the system is not chaotic for some values of the parameters (see figures, Figs 22 and 23) then the plot is periodic.

### 4.2 Non-existence global first integral

In this subsection, we study existence and non-existence first integrals of system (2), we note that if *d* = −7, *r* = 4 and *l* = −1 we have chaos system (1), see [20], we want to show that system (2) has no first integrals in a neighbouhood of singuler points under some conditions.

This section is divided in two subsection. In first section, we study global first integral of system (2), and second one is devoted to study an analytic first integrals.

It appears that you’re referring to a specific subsection discussing the study of a global integral of motion for a system labeled as (2). I understand that the main result of the subsection you’re referring to is the proof that system (2) does not possess a global first integral under certain conditions.

**Theorem 8**. *System* (2) *has no global C*^1^ *first integrals for one of the following conditions holds*.

*i) If* $-\frac{w(bc+d)}{b}>0$ , $d>\;\frac{2b^{3}lr}{cw^{2}-b}$ *and* $2\;blrw+2\;cdw<\frac{w\left(bc+d\right)\left(2\;b^{3}lr-cdw^{2}+bd\right)}{b^{2}}$.
*ii) If* $\frac{w(bc+d)}{b}>0$ , $d>\;\frac{2b^{3}lr}{cw^{2}-b}$ *and* $\frac{w\left(bc+d\right)\left(2\;b^{3}lr-cdw^{2}+bd\right)}{b^{2}}<2\;blrw+2\;cdw$. *Where* $w=\sqrt{-\frac{br(b^{2}l+a)}{blr+cd}}$, *b* ≠ 0 *and* $c\neq -\frac{blr}{d}$.

*Proof*. Since system (2) has two singular points which are $s_{1}=\left(w,-b,\;\frac{dw}{br}\right)$ and $s_{2}=\left(-w,-b,-\;\frac{dw}{br}\right)$, where $w=\sqrt{-\frac{br(b^{2}l+a)}{blr+cd}}$, *b* ≠ 0 and $c\neq -\frac{blr}{d}$. Then eigenvalues of Jacobian matrix of system (2) at the singular pints *s*_1_ and *s*_2_ are zeros of characteristic equations

$$\begin{array}{c} l^{3}-\frac{w\left(bc+d\right)l^{2}}{b}-\frac{(2\;b^{3}lr-cdw^{2}+db)l}{b}+2\;blrw+2\;cdw=0, \end{array}$$ (6)

and

$$\begin{array}{c} l^{3}+\frac{w\left(bc+d\right)l^{2}}{b}-\frac{(2\;b^{3}lr-cdw^{2}+db)l}{b}-2\;blrw-2\;cdw=0, \end{array}$$ (7)

respectively. Then, by Theorem 4 all eigenvalues associated to system (2) at the singular pints $s_{1}=\left(w,-b,\;\frac{dw}{br}\right)$ and $s_{2}=\left(-w,-b,-\;\frac{dw}{br}\right)$, have negative real part if and only if $-\frac{w(bc+d)}{b}>0$, $d>\;\frac{2b^{3}lr}{cw^{2}-b}$ and $2\;blrw+2\;cdw<\frac{w\left(bc+d\right)\left(2\;b^{3}lr-cdw^{2}+bd\right)}{b^{2}}$, for *s*_1_ and $\frac{w(bc+d)}{b}>0$, $d>\;\frac{2b^{3}lr}{cw^{2}-b}$ and $\frac{w\left(bc+d\right)\left(2\;b^{3}lr-cdw^{2}+bd\right)}{b^{2}}<2\;blrw+2\;cdw$, for *s*_2_. Then by Theorem 2, the result is obtained. Thus statement is hold. From Theorem 8, we obtain directly the following result:

**Corollary 1**. *System* (1) *has no global C*^1^ *first integrals for one of the following conditions holds*.

*i) If* $\;\frac{2w(bc-7)}{\sqrt{b}}>0$ , 28*cw*^2^ < 8*b*^2^ + 7 and $16\;b^{3/2}w+28\;c\sqrt{b}w<\;\frac{2w\left(bc-7\right)\left(-28\;cw^{2}+8\;b^{2}+7\right)}{\sqrt{b}}$.

*ii) If* $-\;\frac{2w(bc-7)}{\sqrt{b}}$ , 28*cw*^2^ < 8*b*^2^ + 7 *and* $\;\frac{2w\left(bc-7\right)\left(-28\;cw^{2}+8\;b^{2}+7\right)}{\sqrt{b}}<16\;b^{3/2}w+28\;c\sqrt{b}w$. Where $w=\frac{\sqrt{a-b^{2}}}{\sqrt{4\;b+7\;c}}$, *b* ≠ 0 *and* $c\neq -\;\frac{4b}{\;7}$.

### 4.3 Non-existence analytic first integral

In this subsection, we use Theorem 3 to study analytic first integral in a neighbourhood of singular points.

**Theorem 9**. *The statement you’ve provided indicates that the system labeled as* (2) *does not possess an analytic first integral in the vicinity of its fixed points, given that certain conditions are satisfied*.

*1. If* $a=\frac{-36\;q^{12}r^{2}-441\;l^{2}q^{6}r^{2}+196\;d^{2}}{196\;lr^{2}q^{2}}$ , $b=-\frac{\;3q^{2}}{2}$ and $c=\;\frac{21w^{2}lr}{3\;rw^{6}+7\;d}$, for $s_{1}=\left(w,-b,\;\frac{dw}{br}\right)$.
*2. If* $a=\frac{-36\;q^{12}r^{2}-441\;l^{2}q^{6}r^{2}+196\;d^{2}}{196\;lr^{2}q^{2}}$ , $b=-\frac{\;3q^{2}}{2}$ and $c=-\;\frac{63q^{2}lr}{9\;q^{6}r-21\;d}$, for $s_{2}=\left(-w,-b,-\;\frac{dw}{br}\right)$.

*Where* $\frac{-6\;drw^{6}+63\;lrw^{4}-14\;d^{2}}{63\;w^{3}rl}\neq 0$ , $\frac{-6\;drw^{6}-63\;lrw^{4}+14\;d^{2}}{63\;w^{3}rl}\neq 0$ *and* q∈R\{0}.

*Proof*. If $a=\frac{-36\;q^{12}r^{2}-441\;l^{2}q^{6}r^{2}+196\;d^{2}}{196\;lr^{2}q^{2}}$, $b=-\frac{\;3q^{2}}{2}$ and $c=\;\frac{21w^{2}lr}{3\;rw^{6}+7\;d}.$, then the eigenvalues of Jacobian matrix of system (2) at singular point *s*_1_ are $l_{1}=l_{2}=l_{3}=\frac{-6\;drw^{6}+63\;lrw^{4}-14\;d^{2}}{63\;w^{3}rl}\neq 0.$ Hence, by Theorem 3, **Case 1**, the linearization system (2) has two independent first integrals which are no polynomials. So it seems you want to emphasize that the system (2) lacks any analytic first integrals at the neighbourhood of the fixed point *s*_1_ by Theorem 1.

If $a=\frac{-36\;q^{12}r^{2}-441\;l^{2}q^{6}r^{2}+196\;d^{2}}{196\;lr^{2}q^{2}}$, $b=-\frac{\;3q^{2}}{2}$ and $c=-\;\frac{63q^{2}lr}{9\;q^{6}r-21\;d}$, then the eigenvalues of Jacobian matrix of system (2) at singular point *s*_2_ are $l_{1}=l_{2}=l_{3}=\frac{-6\;drw^{6}-63\;lrw^{4}+14\;d^{2}}{63\;w^{3}rl}\neq 0.$ Hence, by Theorem 3, **Case 1**, the linearization system (2) has two independent first integrals which are no polynomials then system (2) has no analytic first integrals at the neighbourhood of the singular point *s*_2_ by Theorem 1. From Theorem 1, we obtain directly the following result:

**Corollary 2**. *System* (1) *has no analytic first integral at the neighbourhood of singular points, if one of the following conditions holds*.

*1. If* $a=\;\frac{4\;q^{8}+36\;q^{6}-49}{16q^{2}}$ , $b=-\frac{\;3q^{2}}{2}$ *and* $c=\;\frac{36q^{2}}{-6\;q^{4}+21},$ for $s_{1}=\left(-2\;\sqrt{b}w,-b,\;\frac{7w}{2\sqrt{b}}\right)$.
*2. If* $a=\;\frac{4\;q^{8}+36\;q^{6}-49}{16q^{2}}$ , $b=-\frac{\;3q^{2}}{2}$ *and* $c=\;\frac{12q^{2}}{\;2q^{4}+7}$, *for* $s_{2}=\left(2\;\sqrt{b}w,-b,-\;\frac{7w}{2\sqrt{b}}\right).$

*Where* $-\;\frac{4\;q^{4}+49}{18q^{3}}\neq 0$ , $-\;\frac{32\;q^{4}+49}{18q^{3}}\neq 0$ *and* q∈R\{0}.

Now we study a formal series first integral in a the neighbourhood of fixed points (0, 0, *α*) for α∈R\{0} of the system (2).

**Theorem 10**. *System* (2) *has a formal series first integral in a the neighbourhood of equilibrium points* (0, 0, *α*) *for* α∈R\{0}, *for a* = *b* = 0, *d* < 0 *and* $-\;\frac{4d}{r^{2}}<\alpha^{2}$. *Moreover system* (2) *has an analytic first integral in a neighbourhood of the equilibrium points* (0, 0, *α*) *for* $\alpha \in \left(-\;\frac{2\sqrt{-d}}{r},\;\frac{2\sqrt{-d}}{r}\right)\backslash \left\{0\;\right\}$.

*Proof*. Since the system (2) has a non-isolated line of equilibrium points (0, 0, *α*) for α∈R\{0}. The characteristic equation of the Jacobian matrix at the singular point (0, 0, *α*) of system (2) is given by λ^3^ − *αλ*^2^*r* − *dλ* = 0, then the eigenvalues of Jacobian matrix are $l_{1}=0,l_{2}=\frac{r\alpha +\sqrt{\alpha^{2}r^{2}+4\;d}}{2}$ and $l_{3}=\frac{r\alpha -\sqrt{\alpha^{2}r^{2}+4\;d}}{2}$. Since λ_2_ λ_3_ = −*d* and ${l_{2}}^{2}=-d>0$, for *α* ≠ 0 and $-\;\frac{4d}{r^{2}}<\alpha^{2}$ then $k_{2}l_{2}+k_{3}l_{3}=-\frac{d(k_{2}+k_{3})}{l_{2}}\neq 0$, for all $k_{2},k_{3}\in Z^{+}⋃\left\{0\right\}$ with 1 ≤ *k*_2_ + *k*_3_.

Now by Theorem 6, we have that system (2) has a formal series first integral in a neighbourhood of (0, 0, *α*) except the origin. But, if $-\;\frac{2\sqrt{-d}}{r}<\alpha <\;\frac{2\sqrt{-d}}{r}$ then $l_{2}=\frac{r\alpha +i\sqrt{-\alpha^{2}r^{2}-4\;d}}{2}$ and $l_{3}=\frac{r\alpha -i\sqrt{-\alpha^{2}r^{2}-4\;d}}{2}$ ∈C, so either all have positive real parts or all have negative real parts. By Theorem 7, we have that system (2) has an analytic first integral in a neighbourhood of the non-isolated equilibrium points (0, 0, *α*). This concludes the proof.

**Corollary 3**. *System* (1) *has a formal series first integral in a the neighbourhood of equilibrium points* (0, 0, *α*) *for* α∈R\{0}, *a* = *b* = 0 *and* $\;\frac{7}{4}<\alpha^{2}$. *Moreover system* (1) *has an analytic first integral in a neighbourhood of the equilibrium points* (0, 0, *α*) *for* $\alpha \in \left(-\;\frac{\sqrt{7}}{2},\;\frac{\sqrt{7}}{2}\right)\backslash \left\{0\;\right\}$.

*Proof*. The proof of Corollary 3 directly from Theorem 10.

From system (2) when *a* = *b*^2^, *l* = −1, *b* ≠ 0 and *d* ≠ 0 and we perform a change of variables from (*x*, *y*, *z*) → (*X*, *Y*, *Z*) using *X* = *x*, *Y* = *y* + *b*, and *Z* = *z*. This transformation shifts the singular point (0, −*b*, 0) to the origin. Consequently, system (2) transforms into:

$$\begin{array}{c} \dot{x}=y,\;\dot{y}=ryz-rbz+dx,\;\dot{z}=-x^{2}-y^{2}+2by+cxz, \end{array}$$ (8)

the system is rewritten in terms of (*x*, *y*, *z*) instead of (*X*, *Y*, *Z*).

By direct computation we obtain the following.

**Lemma 1**. *The linear part of the system* (8) *exhibits two independent polynomial first integrals at the origin* −2*bx* + *z* *and* (−2*b*^2^*r* − *d*)*x*^2^ + 2*xzbr* + *y*^2^.

**Theorem 11**. Eq (8) *does not possess local analytic first integrals at the singular points* (0, 0, 0); *therefore, it also lacks global analytic first integrals*.

*Proof*. Let’s assume *F* = *F*(*x*, *y*, *z*) represents a local analytic first integral at the origin of system (8). We express it as *F* = ∑_*i* ≥ 0_*F*_*i*_(*x*, *y*, *z*), where *F*_*i*_ denotes a homogeneous polynomial of degree *i* for *i* ≥ 0. We aim to demonstrate this by employing an inductive approach.

$$\begin{array}{c} F_{i}\left(x,y,z\right)=0\;\;\;for\;all\;\;\;i\geq 1. \end{array}$$ (9)

Consequently, we will conclude that *F* = *F*_*i*_. Therefore, *F* would remain constant, contradicting the stipulation that *F* serves as a first integral. Thus, system (8) cannot possess a local analytic first integral at the origin. Next, we will proceed to prove (9). Given that *F* is a first integral of system (8), it is necessary for it to satisfy.

$$\begin{array}{c} y\frac{\partial F}{\partial x}+\left(ryz-rbz+dx\right)\frac{\partial F}{\partial y}+\left(-x^{2}-y^{2}+2by+cxz\right)\frac{\partial F}{\partial z}=0. \end{array}$$ (10)

The terms that involve the variables *x*, *y*, and *z* raised to the power of one in Eq (10) are

$$\begin{array}{c} y\frac{\partial F_{1}}{\partial x}+\left(-rbz+dx\right)\frac{\partial F_{1}}{\partial y}+2by\frac{\partial F_{1}}{\partial z}=0. \end{array}$$ (11)

Hence, *F*_1_ is either equal to zero or a polynomial first integral of degree one derived from the linear part of system (8). By Lemma 1 we get that $F_{1}=c_{0}\left(-2\;bx+z\right)$ with $c_{0}\in R$. Upon computing the terms of degree two in the variables *x*, *y*, and *z* from Eq (10), we obtain

$$\begin{array}{c} y\frac{\partial F_{2}}{\partial x}+\left(-rbz+dx\right)\frac{\partial F_{2}}{\partial y}+2by\frac{\partial F_{2}}{\partial z}+ryz\frac{\partial F_{1}}{\partial y}+\left(-x^{2}-y^{2}+cxz\right)\frac{\partial F_{1}}{\partial z}=0. \end{array}$$ (12)

We assume that $F_{2}=B_{1}x^{2}+B_{2}xy+B_{3}xz+B_{4}y^{2}+B_{5}yz+B_{6}z^{2}$ and substitution *F*_1_ and *F*_2_ in Eq (12), we have that *c*_0_ = 0, and thus *F*_1_ = 0. This proves (9) for *i* = 1. The solution is $F_{2}=c_{1}{H_{1}}^{2}+c_{2}H_{2}$ where $c_{1},c_{2}\in C$, $H_{1}=-2\;bx+z$ and $H_{2}=\left(-2\;b^{2}r-d\right)x^{2}+2\;xzbr+y^{2}$.

We now make the assumption that (9) is valid for *i* = 1, …, *l*_1_ − 1, and we aim to demonstrate its validity for *i* = *l*_1_. Utilizing the induction hypothesis, when computing the terms of degree *l*_2_ in (10), we obtain

$$\begin{array}{c} y\frac{\partial F_{l_{1}}}{\partial x}+\left(-rbz+dx\right)\frac{\partial F_{l_{1}}}{\partial y}+2by\frac{\partial F_{l_{1}}}{\partial z}=0. \end{array}$$ (13)

After establishing that $F_{l_{1}}$ constitutes a non-zero polynomial first integral of degree *l*_2_ associated with the linear portion of system (8), according to Lemma 1, it must adhere to the structure $F_{l_{1}}=F_{l_{1}}\left(H_{1},H_{2}\right)=c_{1}{H_{1}}^{2}+c_{2}H_{2}$.

Subsequently, upon computing the terms of degree *1*_1_ + 1 in (10), we ascertain

$$\begin{array}{c} \begin{array}{cc} & y\frac{\partial F_{l_{1}+1}}{\partial x}+\left(-rbz+dx\right)\frac{\partial F_{l_{1}+1}}{\partial y}+2by\frac{\partial F_{l_{1}+1}}{\partial z}+\left(ryz\right)\left(2y\frac{\partial F_{l_{1}}}{\partial H_{2}}\right)\\ & +\left(-x^{2}-y^{2}+cxz\right)\left(\frac{\partial F_{l_{1}}}{\partial H_{2}}+2\;brx\frac{\partial F_{l_{1}}}{\partial H_{2}}\right)=0. \end{array} \end{array}$$ (14)

If we introduce the notation $F_{l_{1}+1}\left(x,y,z\right)=\psi_{l+1}\left(H_{1},H_{2},z\right)$ with $x=\;\frac{z-H_{1}}{2b}$ and $y=\sqrt{\left(2\;b^{2}r+d\right)x^{2}-2\;brxz+H_{2}}$. So Eq (14) can be written as

$$\begin{array}{c} \begin{array}{cc} \frac{\partial }{\partial z}\psi_{l_{1}+1}\left(H_{1},H_{2},z\right) & =\;\frac{G(H_{1},H_{2},z)}{4\sqrt{{\left(z-H_{1}\right)}^{2}d-2\;b^{2}\left(rz^{2}-r{H_{1}}^{2}-2\;H_{2}\right)}b^{2}}, \end{array} \end{array}$$ (15)

where

$$\begin{array}{cc} G(H_{1},H_{2},z) & =(\left(-2\;rz^{2}+2\;r{H_{1}}^{2}+4\;H_{2}\right)b^{2}-2\;c\left(z-H_{1}\right)zb\\ & +{\left(z-H_{1}\right)}^{2}\left(d+1\right))\frac{\partial }{\partial H_{1}}F_{l_{1}}\left(H_{1},H_{2}\right)\\ & +2\;\left(-cz{\left(z-H_{1}\right)}^{2}b-1/2\;{\left(z-H_{1}\right)}^{2}\left(\left(d+1\right)H_{1}+z\left(d-1\right)\right)\right)r\frac{\partial }{\partial H_{2}}F_{l_{1}}\left(H_{1},H_{2}\right)\\ & +2\;\left(z+H_{1}\right)\left(rz^{2}-r{H_{1}}^{2}-2\;H_{2}\right)b^{2}r\frac{\partial }{\partial H_{2}}F_{l_{1}}\left(H_{1},H_{2}\right). \end{array}$$

Solving (15), we have

$$\begin{array}{cc} \psi_{l_{1}+1}\left(H_{1},H_{2},z\right) & =\;\frac{\eta_{1}\left(H_{1},H_{2}\right)\frac{\partial }{\partial H_{1}}F_{l_{1}}\left(H_{1},H_{2}\right)+\eta_{2}\left(H_{1},H_{2}\right)\frac{\partial }{\partial H_{2}}F_{l_{1}}\left(H_{1},H_{2}\right)}{2{\left(2\;b^{2}r-d\right)}^{7/2}}KK\\ & +\frac{K_{1}\left(H_{1},H_{2},z\right)\left(\eta_{3}\left(H_{1},z\right)\frac{\partial }{\partial H_{1}}F_{l_{1}}\left(H_{1},H_{2}\right)+\eta_{4}\left(H_{1},H_{2},z\right)\frac{\partial }{\partial H_{2}}F_{l_{1}}\left(H_{1},H_{2}\right)\right)}{{\left(2\;b^{2}r-d\right)}^{3}b^{2}}\\ & +k_{2}\left(H_{1},H_{2}\right), \end{array}$$

where

$$\begin{array}{c} \begin{array}{c} KK=\text{arctan}(\frac{2\;b^{2}rz-dz+H_{1}d}{\sqrt{2\;b^{2}r-d}K_{1}\left(H_{1},H_{2},z\right)}), \end{array} \end{array}$$

$$\begin{array}{c} \begin{array}{c} K_{1}\left(H_{1},H_{2},z\right)=\sqrt{{\left(z-H_{1}\right)}^{2}d-2\;b^{2}\left(rz^{2}-r{H_{1}}^{2}-2\;H_{2}\right)}, \end{array} \end{array}$$

$$\begin{array}{c} \begin{array}{cc} \eta_{1}\left(H_{1},H_{2}\right) & =4\;b\left(r^{2}b^{3}-b^{2}rc-1/2\;r\left(d-3\right)b-cd\right)\left(b^{2}r-d/2\right)r{H_{1}}^{2}\\ & +8\;\left(b^{2}r-bc-d/2+1/2\right){\left(b^{2}r-d/2\right)}^{2}H_{2}, \end{array} \end{array}$$

$$\begin{array}{c} \begin{array}{cc} \eta_{2}\left(H_{1},H_{2}\right) & =-\left(4\;b^{4}r^{2}-8\;b^{3}cr-2\;\left(3\;d-5\right)rb^{2}-6\;bcd+2\;d^{2}\right)b^{2}r^{3}{H_{1}}^{3}\\ & -\left(8\;b^{4}r^{2}-16\;b^{3}cr-12\;r\left(d-1\right)b^{2}-4\;bcd+4\;d^{2}\right)\left(b^{2}r-d/2\right)H_{2}rH_{1}, \end{array} \end{array}$$

$$\begin{array}{c} \begin{array}{cc} \eta_{3}\left(H_{1},z\right) & =\left(b^{2}r-d/2\right)\left(-2\;\left(bc-d/4-1\right)b^{2}r-1/2\;\left(bc+d/2+1/2\right)d\right)H_{1}\\ & +z\left(b^{2}r+bc-d/2-1/2\right){\left(b^{2}r-d/2\right)}^{2}, \end{array} \end{array}$$

and

$$\begin{array}{cc} \eta_{4}\left(H_{1},H_{2},z\right) & =\left(2/3\;b^{6}{H_{1}}^{2}-zb^{6}H_{1}-2/3\;b^{6}z^{2}\right)r^{4}\\ & +\left(10/3\;b^{4}\left(bc-d/4-\frac{11}{10}\right){H_{1}}^{2}-\left(2\;z\left(bc-\frac{5\;d}{12}-3/4\right)b^{4}-1/2\;zb^{4}d\right)H_{1}\right)r^{3}\\ & +\left(-2/3\;\left(-b^{2}dz^{2}+\left(-b^{3}c+1/2\;b^{2}\right)z^{2}-2\;b^{4}H_{2}\right)b^{2}+1/3\;b^{4}z^{2}d\right)r^{3}\\ & +(b^{2}d\left(bc+\frac{7\;d}{12}+3/4\right){H_{1}}^{2}-(-1/6\;zd\left(bc-d-2\right)b^{2}\\ & -z\left(bc-\frac{5\;d}{12}-3/4\right)b^{2}d)H_{1})r^{2}\\ & -2/3\;\left(1/4\;d^{2}z^{2}+\left(\left(1/2\;bc-1/4\right)z^{2}+H_{2}b^{2}\right)d-4\;b^{3}cH_{2}+2\;H_{2}b^{2}\right)b^{2}r^{2}\\ & +1/3\;\left(-b^{2}dz^{2}+\left(-b^{3}c+1/2\;b^{2}\right)z^{2}-2\;b^{4}H_{2}\right)dr^{2}\\ & +(-1/12\;d^{2}\left(bc+2\;d+1\right){H_{1}}^{2}-1/12\;zd^{2}\left(bc-d-2\right)H_{1})r\\ & +1/3\;(1/4\;d^{2}z^{2}+\left(\left(1/2\;bc-1/4\right)z^{2}+H_{2}b^{2}\right)d-4\;b^{3}cH_{2}+2\;H_{2}b^{2})dr, \end{array}$$

where *K*_2_ is a function in the variables *H*_1_ and *H*_2_. Since $F_{l_{1}+1}$ must be a polynomial, so $\eta_{1}\left(H_{1},H_{2}\right)=\eta_{2}\left(H_{1},H_{2}\right)=\eta_{3}\left(H_{1},z\right)=\eta_{4}\left(H_{1},H_{2},z\right)=0$, we have *d* = 0 is contradiction to the hypothesis, we get that $\frac{\partial }{\partial H_{1}}F_{l_{1}}\left(H_{1},H_{2}\right)=\frac{\partial }{\partial H_{2}}F_{l_{1}}\left(H_{1},H_{2}\right)=0$. Given that $F_{l_{1}}$ possesses a degree of *l*_2_, it follows that $F_{l_{1}}=0.$ This confirmation establishes (9) for *i* = *l_i_*. Consequently, this validates (9) overall, thereby confirming the proof of Theorem 11. When *c* = 0 and *l* = −1, the system represented by (2) transforms to

$$\begin{array}{c} \dot{x}=y+b,\;\dot{y}=dx+ryz,\;\dot{z}=a-x^{2}-y^{2}, \end{array}$$ (16)

To find the equilibrium points of system (16), we identify them as follows: *E*_0_ = (0, −*b*, 0), if *a* = *b*^2^, $E_{1}=\left(\sqrt{a-b^{2}},-b,\;\frac{d\sqrt{a-b^{2}}}{br}\right)$ and $E_{2}=\left(-\sqrt{a-b^{2}},-b,-\;\frac{d\sqrt{a-b^{2}}}{br}\right)$ with being real when *a* − *b*^2^ ≥ 0. Next, we will analyze the presence or absence of analytic first integrals in system (16).

**Lemma 2**. *If a* = *b*^2^, *b* ≠ 0, *linearized system* (16) *at E*_0_ = (0, −*b*, 0) *has two linear independent polynomial first integrals*.

*Proof*. If *a* = *b*^2^, *b* ≠ 0 then system (16) we have only the equilibrium point *E*_0_ = (0, −*b*, 0), We perform a change of variables from (*x*, *y*, *z*) → (*X*, *Y*, *Z*) using *X* = *x*, *Y* = *y* + *b*, *Z* = *z*. This transformation shifts the singular point *E*_0_ = (0, −*b*, 0) to the origin. Consequently, system (16) transforms into:

$$\begin{array}{c} \dot{x}=y,\;\dot{y}=dx+ryz-brz,\;\dot{z}=2by-x^{2}-y^{2}, \end{array}$$ (17)

In the expression, we have reverted to using (*x*, *y*, *z*) instead of (*X*, *Y*, *Z*). The linear part of system (17) at the origin is:

$$\begin{array}{c} \dot{x}=y,\;\dot{y}=dx-brz,\;\dot{z}=2by, \end{array}$$ (18)

easily by direct computations from definition of first integral shows

$$\begin{array}{c} y\frac{\partial }{\partial x}F_{i}\left(x,y,z\right)+\left(dx-brz\right)\frac{\partial }{\partial y}F_{i}\left(x,y,z\right)+2by\;\left(\frac{\partial }{\partial z}F_{i}\left(x,y,z\right)\right)=0, \end{array}$$ (19)

where *H*_1_(*x*, *y*, *z*) = −2*bx* + *z* and *H*_2_(*x*, *y*, *z*) = (−2*rb*^2^ −*d*)*x*^2^ + *zrxb* + *y*^2^.

Hence, the function *F_i_* remains constant across the solutions of system (17) for *i* = 1, 2. Given that the linear part of system (17) at the origin possesses two distinct independent polynomial first integrals, we can demonstrate that system (17) lacks local analytic first integrals using a similar approach as employed in the proof of Theorem 11.

Because the system (16) is symmetric to (*x*, *y*, *z*, *t*) → (−*x*, *y*, −*z*, −*t*), we study analytic first integral only for the equilibrium point $E_{1}=\left(\sqrt{a-b^{2}},-b,\;\frac{d\sqrt{a-b^{2}}}{br}\right)$.

The transform of variables (*x*, *y*, *z*) → (*X*, *Y*, *Z*) given by $X=x-\sqrt{a-b^{2}},Y=y+b,Z=z-\frac{d\sqrt{a-b^{2}}}{br}$ move to equilibrium point $E_{1}=\left(\sqrt{a-b^{2}},-b,\;\frac{d\sqrt{a-b^{2}}}{br}\right)$ to an equilibrium point at the origin and system (16) becomes

$$\begin{array}{c} \dot{x}=y,\;\dot{y}=dx+\frac{d\sqrt{a-b^{2}}}{b}y-brz+ryz,\;\dot{z}=-2\;\sqrt{a-b^{2}}x+2\;by-x^{2}-y^{2}, \end{array}$$ (20)

The Jacobian matrix of system (20) calculated at (0, 0, 0) is

$$\begin{array}{c} J=(\begin{array}{ccc} 0 & 1 & 0\\ d & \frac{d\sqrt{a-b^{2}}}{b} & -br\\ -2\;\sqrt{a-b^{2}} & 2\;b & 0 \end{array}). \end{array}$$

The characteristic equation of the matrix *J* is represented by

$$\begin{array}{c} u^{3}-\frac{d\sqrt{a-b^{2}}u^{2}}{b}-\left(-2\;b^{2}r+d\right)u-2\;br\sqrt{a-b^{2}}=0. \end{array}$$ (21)

**Lemma 3**. *The characteristic equation, labeled as* (21), *exhibits a single unique real root denoted as* λ *and a pair of complex roots*, *α*±*i β*, *where* λ, *α*, *and β* *belong to the set of real numbers*.

$$\begin{array}{c} l+2\;\alpha =\frac{d\sqrt{a-b^{2}}}{b},\;\;\alpha^{2}+2\;l\;\alpha +\beta^{2}=2\;b^{2}r-d\;\;and\;\;l\;\left(\alpha^{2}+\beta^{2}\right)=2\;br\sqrt{a-b^{2}}. \end{array}$$ (22)

*Proof*. The characteristic Eq (21) can be rewritten

$$\begin{array}{c} u^{3}-\frac{d\sqrt{a-b^{2}}u^{2}}{b}-\left(-2\;b^{2}r+d\right)u-2\;br\sqrt{a-b^{2}}=\left(u-l\right)\left(u-\alpha -i\beta \right)\left(u-\alpha +i\beta \right), \end{array}$$

By comparing the above equation and Eq (21) we obtain the conditions (22).

**Theorem 12**. *If* $a\neq -b^{2}m,r\neq \;\frac{4\;\beta^{2}m^{2}-m-1}{8b^{2}m^{2}},$ *and* $d\neq \frac{m+1}{m}$ or *a* ≠ *b*^2^, $d\neq \frac{m^{2}}{\left(m-1)(m+1\right)}$ *and* $r\neq \;\frac{m^{4}}{2\left(m-1\right)\left(m+1\right)b^{2}}$, m∈Z\{-1,0,1}. *Hence, it follows that with in a neighborhood of the equilibrium point* $E_{1}=\left(\sqrt{a-b^{2}},-b,\;\frac{d\sqrt{a-b^{2}}}{br}\right)$, *system* (16) *does not possess a local analytic first integral*.

*Proof*. If $a\neq -b^{2}m,r\neq \;\frac{4\;\beta^{2}m^{2}-m-1}{8b^{2}m^{2}},$ and $d\neq \frac{m+1}{m}$, l,α,β∈R\{0}, satisfies the conditions of Lemma 3, the characteristic Eq (21) displays a single distinct real root denoted by λ and two complex roots *α*±i *β*. By Theorem 3 is the one given in **Case 2**, then only the expression *F*_1_ constitutes a polynomial first integral if and only if either one of the following conditions is satisfied: λ = 2*αm* or *α* = 0 and *β* = *m*, where *α* is positive integer and *m* is negative integer (or *m* is positive integer and *α* is negative integer because ${F_{1}}^{-1}$ is also the first integral), in the context of the first case, when λ, *α* and *β* comply with Eq (22), it implies that, so substitution λ = 2*αm*, in Eq (22), we obtain the solution $a=-b^{2}m,r=\;\frac{4\;\beta^{2}m^{2}-m-1}{8b^{2}m^{2}},$ and $d=\frac{m+1}{m}$. By the hypothesis none of them are possible.

In the second case, similarly considering that λ, *α* and *β* must satisfy Eq (22), this implies, so substitution *α* = 0 and *β* = *m*, in Eq (22), we obtain the solution *a* = *b*^2^, $d=\frac{m^{2}}{\left(m-1)(m+1\right)}$ and $r=\;\frac{m^{4}}{2\left(m-1\right)\left(m+1\right)b^{2}}$, which is obviously not possible. Therefore, the linear part of system (20) has no polynomial first integrals. Then, directly using Theorem 1 we can say that system (16) has no local analytic first integral at the neighborhood of the equilibrium point *E*_1_.

## 5 Conclusion

In the present work, we have successfully developed a new 3-D chaotic system characterized by three Lyapunov exponents: one positive, one zero, and one negative. The chaotic nature of the model is evident through the depiction of phase trajectories, illustration of bifurcation patterns, and visualization of Lyapunov exponent graphs. These findings confirm the dynamic complexity and chaotic behavior inherent in the proposed 3-D chaotic system. We explore both local and global analytic first integrals for the system, providing results on the existence and non-existence of these integrals for different parameter values. Our findings indicate that the system lacks a global first integral, and the presence or absence of analytic first integrals depends on specific parameter values. Additionally, we present a formal series for the system. Furthermore, we demonstrate 3D and 2D projections of the system (2) with self-excited and hidden attractors for a given set of initial conditions by selecting alternative values for parameters *a*, *c*, *d*, *r*, and *l*.

## References

[1] Sprott JC. Simple chaotic systems and circuits. American Journal of Physics. 2000;68(8):758–763. doi: 10.1119/1.19538

[2] Xiaofu L, Aubrey B, Robert D, Perkins E. Chaos in a pendulum adaptive frequency oscillator circuit experiment. Chaos Theory and Applications;5(1):11–19.

[3] Majid Sheikh Zain, Asjad Muhammad Imran, Faridi Waqas Ali. Solitary travelling wave profiles to the nonlinear generalized Calogero–Bogoyavlenskii–Schiff equation and dynamical assessment. Eur Phys J Plus. 2023;138(11):1040. doi: 10.1140/epjp/s13360-023-04681-z

[4] Alqurashi NT, Manzoor M, Majid SZ, Asjad MI, Osman M. Solitary waves pattern appear in tropical tropospheres and mid-latitudes of nonlinear Landau-Ginzburg-Higgs equation with chaotic analysis. Results in Physics. 2023;54:107116. doi: 10.1016/j.rinp.2023.107116

[5] Hasler MJ. Electrical circuits with chaotic behavior. Proceedings of the IEEE. 1987;75(8):1009–1021. doi: 10.1109/PROC.1987.13846

[6] Chua LO, Wu CW, Huang A, Zhong GQ. A universal circuit for studying and generating chaos. I. Routes to chaos. IEEE Transactions on Circuits and Systems I: Fundamental Theory and Applications. 1993;40(10):732–744. doi: 10.1109/81.246150

[7] Nakagawa S, Saito T. An RC OTA hysteresis chaos generator. In: 1996 IEEE International Symposium on Circuits and Systems (ISCAS). vol. 3. IEEE; 1996. p. 245–248.

[8] Tamaševičius A, Namajunas A, Čenys A. Simple 4D chaotic oscillator. Electronics Letters. 1996;32(11):957–958. doi: 10.1049/el:19960630

[9] Ogorzalek MJ. Order and chaos in a third-order RC ladder network with nonlinear feedback. IEEE Transactions on circuits and systems. 1989;36(9):1221–1230. doi: 10.1109/31.34668

[10] Kawakami H. Bifurcation of periodic responses in forced dynamic nonlinear circuits: Computation of bifurcation values of the system parameters. IEEE Transactions on circuits and systems. 1984;31(3):248–260. doi: 10.1109/TCS.1984.1085495

[11] Saito T. Chaotic phenomena in a coupled oscillators. In: Proceedings of European Conf. on Circuit Theory and Design; 1987. p. 275–280.

[12] Çiçek S, Ferikoğlu A, Pehlivan I. A new 3D chaotic system: dynamical analysis, electronic circuit design, active control synchronization and chaotic masking communication application. Optik. 2016;127(8):4024–4030. doi: 10.1016/j.ijleo.2016.01.069

[13] Llibre J, Valls C. Formal and analytic integrability of the Lorenz system. Journal of Physics A: Mathematical and General. 2005;38(12):2681. doi: 10.1088/0305-4470/38/12/010

[14] Llibre J, Zhang X. Darboux integrability for the Rössler system. International Journal of Bifurcation and Chaos. 2002;12(02):421–428. doi: 10.1142/S0218127402004474

[15] Zhang X. Exponential factors and Darboux integrability for the Rössler system. International Journal of Bifurcation and Chaos. 2004;14(12):4275–4283. doi: 10.1142/S0218127404011922

[16] Lăzureanu C. Integrable deformations of three-dimensional chaotic systems. International Journal of Bifurcation and Chaos. 2018;28(05):1850066. doi: 10.1142/S0218127418500669

[17] Sambas A, Vaidyanathan S, Zhang X, Koyuncu I, Bonny T, Tuna M, et al. A novel 3D chaotic system with line equilibrium: multistability, integral sliding mode control, electronic circuit, FPGA implementation and its image encryption. IEEE Access. 2022;10:68057–68074. doi: 10.1109/ACCESS.2022.3181424

[18] Sambas A, Miroslav M, Vaidyanathan S, Ovilla-Martínez B, Tlelo-Cuautle E, Abd El-Latif AA, et al. A New Hyperjerk System with a Half Line Equilibrium: Multistability, Period Doubling Reversals, Antimonotonocity, Electronic Circuit, FPGA Design and an Application to Image Encryption. IEEE Access. 2024;.

[19] Benkouider K, Vaidyanathan S, Sambas A, Tlelo-Cuautle E, Abd El-Latif AA, Abd-El-Atty B, et al. A New 5-D multistable hyperchaotic system with three positive lyapunov exponents: bifurcation analysis, circuit design, FPGA realization and image encryption. IEEE Access. 2022;10:90111–90132. doi: 10.1109/ACCESS.2022.3197790

[20] Rajagopal K, Akgul A, Jafari S, Karthikeyan A, Koyuncu I. Chaotic chameleon: Dynamic analyses, circuit implementation, FPGA design and fractional-order form with basic analyses. Chaos, Solitons &Fractals. 2017;103:476–487. doi: 10.1016/j.chaos.2017.07.007

[21] Jalal AA, Amen AI, Sulaiman NA. Darboux integrability of the simple chaotic flow with a line equilibria differential system. Chaos, Solitons &Fractals. 2020;135:109712. doi: 10.1016/j.chaos.2020.109712

[22] Bragin V, Vagaitsev V, Kuznetsov N, Leonov G. Algorithms for finding hidden oscillations in nonlinear systems. The Aizerman and Kalman conjectures and Chua’s circuits. Journal of Computer and Systems Sciences International. 2011;50:511–543. doi: 10.1134/S106423071104006X

[23] Leonov GA, Kuznetsov NV. Hidden attractors in dynamical systems. From hidden oscillations in Hilbert-Kolmogorov, Aizerman, and Kalman problems to hidden chaotic attractor in Chua circuits. International Journal of Bifurcation and Chaos. 2013;23(01):1330002. doi: 10.1142/S0218127413300024

[24] Leonov G, Kuznetsov N, Mokaev T. Homoclinic orbits, and self-excited and hidden attractors in a Lorenz-like system describing convective fluid motion: Homoclinic orbits, and self-excited and hidden attractors. The European Physical Journal Special Topics. 2015;224:1421–1458. doi: 10.1140/epjst/e2015-02470-3

[25] Falconi M, Llibre J. Hamiltonian theory of integrability and linear differential equations. Qual Theory Dyn Syst. 2004;4:233–254. doi: 10.1007/BF02970860

[26] Llibre J, Messias M, Da Silva PR. On the global dynamics of the Rabinovich system. Journal of Physics A: Mathematical and Theoretical. 2008;41(27):275210. doi: 10.1088/1751-8113/41/27/275210

[27] Lima MF, Llibre J, Valls C. Integrability of the Rucklidge system. Nonlinear Dynamics. 2014;77:1441–1453. doi: 10.1007/s11071-014-1389-y

[28] Llibre J, Valls C. Liouvillian first integrals of quadratic-linear polynomial differential systems. Journal of mathematical analysis and applications. 2011;379(1):188–199. doi: 10.1016/j.jmaa.2010.12.033

[29] Zhang X. Regularity and convergence of local first integrals of analytic differential systems. Journal of Differential Equations. 2021;294:40–59. doi: 10.1016/j.jde.2021.05.018

[30] Oliveira RD, Rezende AC. Global phase portraits of a SIS model. Applied Mathematics and Computation. 2013;219(9):4924–4930. doi: 10.1016/j.amc.2012.10.090

[31] Oliveira R, Valls C. Global dynamical aspects of a generalized Chen-Wang differential system. Nonlinear Dynamics. 2016;84:1497–1516. doi: 10.1007/s11071-015-2584-1

[32] Rama Mohana Rao M. Ordinary differential equations: theory and applications. (No Title). 1980;.

[33] Llibre J, Saghin R, Zhang X. On the analytic integrability of the 5-dimensional Lorenz system for the gravity-wave activity. Proceedings of the American Mathematical Society. 2014;142(2):531–537. doi: 10.1090/S0002-9939-2013-11773-9

[34] Li W, Llibre J, Zhang X. Local first integrals of differential systems and diffeomorphisms. Zeitschrift für angewandte Mathematik und Physik ZAMP. 2003;54:235–255. doi: 10.1007/s000330300003

[35] Li C, Sprott J. Finding coexisting attractors using amplitude control. Nonlinear Dynamics. 2014;78:2059–2064. doi: 10.1007/s11071-014-1568-x

[36] Zhang X. A note on local integrability of differential systems. Journal of Differential Equations. 2017;263(11):7309–7321. doi: 10.1016/j.jde.2017.08.016

[37] Wolf A, Swift JB, Swinney HL, Vastano JA. Determining Lyapunov exponents from a time series. Physica D: nonlinear phenomena. 1985;16(3):285–317. doi: 10.1016/0167-2789(85)90011-9

[38] Kaplan JL, Yorke JA. Chaotic behavior of multidimensional difference equations. In: Functional Differential Equations and Approximation of Fixed Points: Proceedings, Bonn, July 1978. Springer; 2006. p. 204–227.

